# SeqBattNet: A discrete-state physics-informed neural network with aging adaptation for battery modeling

**DOI:** 10.1371/journal.pone.0362362

**Published:** 2026-11-12

**Authors:** Khoa Tran, Hung-Cuong Trinh, Vy-Rin Nguyen, T. Nguyen-Thoi, Vin Nguyen-Thai, Hung Pham, Ho-Si-Hung Nguyen

**Affiliations:** 1 Data Science Laboratory, Faculty of Information Technology, Ton Duc Thang University, Ho Chi Minh City, Vietnam; 2 Natural Language Processing and Knowledge Discovery Research Group, Faculty of Information Technology, Ton Duc Thang University, Ho Chi Minh City, Vietnam; 3 Department of Semiconductor and Microelectronics, FPT University, Da Nang, Vietnam; 4 Laboratory for Applied and Industrial Mathematics, Institute for Computational Science and Artificial Intelligence, Van Lang University, Ho Chi Minh City, Vietnam; 5 Faculty of Mechanical, Electrical, and Computer Engineering, Van Lang School of Technology, Van Lang University, Ho Chi Minh City, Vietnam; 6 Laboratory for Computational Mechanics, Institute for Computational Science and Artificial Intelligence, Van Lang University, Ho Chi Minh City, Vietnam; 7 Faculty of Civil Engineering, Van Lang School of Technology, Van Lang University, Ho Chi Minh City, Vietnam; 8 School of Science, Engineering & Technology, RMIT, Ho Chi Minh City, Vietnam; 9 Faculty of Electrical Engineering, The University of Danang - University of Science and Technology, Da Nang, Vietnam; National Changhua University of Education, TAIWAN

## Abstract

Accurate battery modeling is essential for reliable state estimation in modern applications, such as predicting the remaining discharge time and remaining discharge energy in battery management systems. Existing approaches face several limitations: model-based methods require a large number of physical parameters; data-driven methods rely heavily on labeled datasets; and current physics-informed neural networks (PINNs) often lack aging adaptation, still depend on many physical parameters, or require continuous state regeneration. In this work, we propose **SeqBattNet**, a discrete-state PINN with built-in aging adaptation for battery modeling to predict terminal voltage during the discharge process. SeqBattNet consists of two components: (i) an encoder, implemented as the proposed Hierarchical Reasoning–GRU deep learning module, which generates cycle-specific aging adaptation parameters; and (ii) a decoder, based on the equivalent circuit model (ECM) combined with deep learning, which uses these parameters together with the input current to predict voltage. The model requires only a small number of readily available physical battery parameters and, when trained on data from a single cell, demonstrates promising predictive performance on unseen battery cells within the evaluated datasets. Experimental results on three benchmark datasets (TRI, RT-Batt, and NASA) show that SeqBattNet consistently outperforms classical sequence models and recent PINN baselines. On the NASA dataset, SeqBattNet reduces the RMSE from 0.0382 to 0.0257 for battery RW4 and from 0.0406 to 0.0253 for battery RW5 compared with the state-of-the-art PDB-Net, corresponding to relative improvements of approximately 32.7% and 37.7%.

## 1 Introduction

In recent years, the use of battery-powered devices—such as electric vehicles, portable electronic devices, and unmanned aerial vehicles—has rapidly increased, highlighting the critical role of batteries in modern technology [1]. Among various battery types, lithium-ion batteries (LIBs) are the most widely used due to their high energy density and long cycle life. The global LIB market is projected to exceed 170 billion USD by 2030 [2]. However, LIBs exhibit strong coupling effects and nonlinear behavior, posing significant challenges for their control and management.

Therefore, an accurate Battery Management System (BMS) [3] is essential for monitoring the health, safety, and performance of batteries. A typical BMS encompasses two core functions: life cycle prognosis and in-cycle state estimation. Life cycle prognosis focuses on long-term performance metrics such as state of health (SOH) [4–7], remaining useful life (RUL) [8–11], and cycle life prediction [2,12]. These aspects have attracted considerable research interest due to their critical role in extending battery lifespan and ensuring system reliability. On the other hand, in-cycle state estimation involves real-time indicators such as state of charge (SOC) [13–15], remaining discharge time (RDT) [1,16,17], and remaining discharge energy (RDE) [17–19], to enhance user experience (range and time-to-empty guidance, reduced range anxiety, charge scheduling) [20].

In approaches to RDT prediction and RDE estimation, battery modeling [21] is a crucial component for predicting terminal voltage from the input current, in order to determine the time at which the predicted voltage falls below the cut-off voltage (the end-of-discharge time), which is then used to estimate the remaining discharge time and, subsequently, the remaining discharge energy. Battery modeling has attracted increasing attention in recent years, particularly in RDT [16,21,22] and RDE estimation [19,23]. These approaches are generally classified into three categories: model-based, data-driven, and physics-informed neural network (PINN) approaches.

Model-based methods characterize battery dynamics and degradation via physics-based formulations, including electrochemical models [24], and ECMs [25]. ECMs approximate a cell with an open-circuit voltage (OCV) source in series with an ohmic resistance and one or more RC polarization branches [26]. Within electrochemical approaches, the pseudo two-dimensional (P2D/DFN) [27] and single-particle model (SPM) [28] are prevalent. Although high-fidelity, these models demand many difficult-to-identify parameters and careful calibration, rendering parameter identification expensive and real-time deployment computationally burdensome [29].

Data-driven methods use machine learning models to learn battery behavior directly from measured data rather than from explicit physical equations. Consequently, they require large, well-labeled datasets spanning diverse operating conditions to capture variability in behavior [22]. proposed *Dynaformer*, which employs an encoder to generate context for the current battery degradation state and passes this context to a decoder that, together with the input current, predicts the terminal voltage. In general, data-driven methods are less commonly used in battery modeling for RDT or RDE estimation, because reliable predictions require large, diverse labeled datasets to generalize across operating conditions. However, generating battery labels is costly and time-consuming [30], and data-driven methods lack interpretability, making them less practical for some battery management systems.

To bridge the gap between model-based and data-driven approaches, physics-informed neural networks (PINNs) have increasingly been applied to battery state estimation and prognosis. Existing physics-informed battery modeling methods can generally be categorized into two directions: (1) neural networks trained with physical constraints incorporated into the loss function, and (2) hybrid architectures that integrate neural networks with explicit battery models, such as equivalent circuit models (ECMs).

As an example of the first direction [31], propose a PINN for SOH estimation by embedding battery degradation dynamics into the training objective. Their model combines a data-fitting loss with a monotonicity constraint and a physics loss, encouraging the predicted SOH trajectory to remain consistent with the long-term ageing behavior of the battery. Although this degradation-oriented formulation is appropriate for SOH estimation, it is not directly aligned with the physical mechanism governing RDT. SOH describes gradual capacity degradation over repeated operating cycles, whereas RDT represents the remaining operating time within a discharge process before the terminal voltage reaches the cut-off threshold. Therefore, RDT prediction depends more directly on short-term electrical dynamics, including the effects of current load, internal resistance, polarization voltage, SOC, and terminal voltage response. For this reason, the second direction, in which a neural network is integrated with an ECM, is more suitable for RDT prediction.

In the second direction, PINNs incorporate governing physical relationships into neural network training, improving physical consistency while reducing dependence on large labeled datasets [19]. proposed a PINN based on an equivalent circuit model (ECM), namely the nonlinear double capacitor (NDC) model. Their method generates battery states ($V_{b}$, $V_{s}$, *V*_1_, *T*_core_, *T*_surf_) by solving ordinary differential equations (ODEs), and combines these states with the current input to predict the terminal voltage using a feedforward neural network. Although continuous-state formulations effectively model short-term battery dynamics, they propagate the hidden states continuously over long prediction horizons. Consequently, modeling errors accumulate over time, making it difficult to accurately capture long-term battery degradation and determine the exact end-of-discharge (EOD) point. Moreover, continuous-state methods generally require a predefined prediction horizon, which may produce voltage trajectories extending beyond the cutoff voltage and lead to overestimation of the remaining discharge time. To address these limitations [21], proposed a discrete-state PINN that updates the battery state sequentially at each time step and terminates prediction once the estimated terminal voltage reaches the cutoff voltage. Such a discrete-state formulation is more suitable for battery prognosis because it naturally follows the stepwise discharge process, allows adaptive termination at EOD, and limits long-term error accumulation. Nevertheless, the model still requires physical parameters such as $R_{p}$, $R_{s}$, $C_{sp}$, and $C_{s}$, which must be identified through experiments or grid search. Furthermore, it does not incorporate aging adaptation, potentially reducing prediction accuracy as the battery degrades. Its evaluation protocol may also introduce data leakage because the first 60 data points of each discharge cycle are used for training while the remaining points from the same cell are used for testing, potentially overestimating the model’s generalization ability.

Recent studies have further explored physics-integrated learning for battery modeling [32]. proposed MINN, which integrates neural networks with electrochemical differential-algebraic equations to learn battery dynamics. More recently [33], introduced BMINN, a Bayesian framework for learning chemical potentials and thermodynamic parameters from voltage data in multiphase battery modeling. Unlike these approaches, SeqBattNet integrates a discrete-state ECM with neural networks and incorporates cycle-specific aging adaptation for terminal voltage prediction.

Despite these advances, reliable battery state estimation and prognosis remain challenging, particularly under battery aging and varying operating conditions [34]. These challenges highlight the need for physically consistent and data-efficient battery prediction models that can account for battery degradation while maintaining predictive accuracy.

Motivated by these limitations, we propose the following contributions:

- We propose **SeqBattNet**, a discrete-state PINN for battery modeling that addresses the limitations of existing approaches: (i) model-based methods, which require numerous physical parameters; (ii) data-driven methods, which typically rely on large training datasets; and (iii) prior PINN baselines, which often adopt continuous ODE formulations that make proactive RDT termination difficult, or require hard-to-determine physical parameters such as $R_{p}$, $R_{s}$, $C_{sp}$, and $C_{s}$. In contrast, SeqBattNet uses readily available battery information, including $V_{OCV,max}$, *V*_EOD_, *C*_rated_, and *C*_EOL_, together with neural-network-predicted adaptive parameters to account for battery ageing. The proposed discrete-state formulation enables sequential terminal voltage prediction under a given current profile. This study evaluates voltage prediction accuracy rather than EOD timing or RDT estimation. Moreover, SeqBattNet was trained on data from a single cell while maintaining strong predictive accuracy on unseen cells within the evaluated datasets. Finally, the model is optimized using a proposed weighted loss function that encourages the predicted voltage to closely match the actual voltage near the end-of-discharge segment.
- The proposed model is validated on three public battery datasets: (i) a dataset with a constant discharge current across all cells and charge-discharge cycles, (ii) a dataset with fixed discharge profiles across cycles within each cell but differing between cells, and (iii) a dataset with random discharge profiles across both cycles and cells. Unlike [21], where training and testing data are taken from the same cell, our data-splitting strategy uses separate cells for training, validation, and testing. This approach provides a more general and fair evaluation of model performance. Under this setting, SeqBattNet outperforms strong baselines. In this work, we do not consider temperature effects.

The rest of this paper is structured as follows. Section 2 introduces the preliminaries. Section 3 presents the proposed approach, including the model architecture and evaluation metrics. Section 4 details the datasets and provides data analysis. Section 5 describes the experimental setup, reports model performance, and discusses future directions. Finally, Section 6 summarizes the findings.

## 2 Preliminaries

**The Gated Recurrent Unit (GRU) [**35**]** is widely used in sequence modeling [36], as it effectively captures temporal dependencies while reducing parameter complexity compared to Long Short-Term Memory (LSTM) networks [37] and Transformers [38]. Given input ${𝐱}_{t}\in {\mathbb{R}}^{d}$ and previous hidden state ${𝐡}_{t-1}\in {\mathbb{R}}^{m}$, a GRU cell updates as:


$$\begin{array}{cccc} {𝐳}_{t} & =\sigma \;\left({𝐖}_{z}{𝐱}_{t}+{𝐔}_{z}{𝐡}_{t-1}+{𝐛}_{z}\right), & & \text{(update gate)}\\ {𝐫}_{t} & =\sigma \;\left({𝐖}_{r}{𝐱}_{t}+{𝐔}_{r}{𝐡}_{t-1}+{𝐛}_{r}\right), & & \text{(reset gate)}\\ {\tilde{𝐡}}_{t} & =\tanh\;\left({𝐖}_{h}{𝐱}_{t}+{𝐔}_{h}\left({𝐫}_{t}⊙{𝐡}_{t-1}\right)+{𝐛}_{h}\right), & & \text{(candidate state)}\\ {𝐡}_{t} & =(1-{𝐳}_{t})⊙{𝐡}_{t-1}+{𝐳}_{t}⊙{\tilde{𝐡}}_{t}, & & \text{(new hidden state)} \end{array}$$ (1)

where σ(·) is the sigmoid function, tanh(·) is the hyperbolic tangent, and ⊙ denotes element-wise multiplication. The variables are defined as follows: ${𝐱}_{t}\in {\mathbb{R}}^{d}$ is the input vector at time step *t*, ${𝐡}_{t-1},{𝐡}_{t}\in {\mathbb{R}}^{m}$ are the previous and current hidden states, ${𝐳}_{t},{𝐫}_{t},{\tilde{𝐡}}_{t}\in {\mathbb{R}}^{m}$ are the update gate, reset gate, and candidate sta*t*e, respectively, ${𝐖}_{\left(\cdot \right)}\in {\mathbb{R}}^{m\times d}$ and ${𝐔}_{\left(\cdot \right)}\in {\mathbb{R}}^{m\times m}$ are learnable weight matrices, and ${𝐛}_{\left(\cdot \right)}\in {\mathbb{R}}^{m}$ are bias terms. The GRU combines the update gate ${𝐳}_{t}$ and reset gate ${𝐫}_{t}$ to control information flow and capture temporal dependencies while mitigating the vanishing-gradient problem. In this work, we use the GRU cell, denoted as GRU(·,·), in the encoder component of our proposed model.

## 3 Proposed method

Our SeqBattNet consists of two main components: an encoder and a decoder, as illustrated in Fig 1. The encoder employs a novel Hierarchical Reasoning–GRU to predict adaptation parameters corresponding to the current battery state. The decoder implements a physics-informed ECM that combines physical equations with neural networks to predict the terminal voltage from the estimated adaptation parameters and the input current. SeqBattNet is trained with a customized loss function that places stronger emphasis on the beginning and the end of the predicted voltage trajectory, thereby guiding the model to better follow the true terminal voltage sequence.

**Fig 1:**
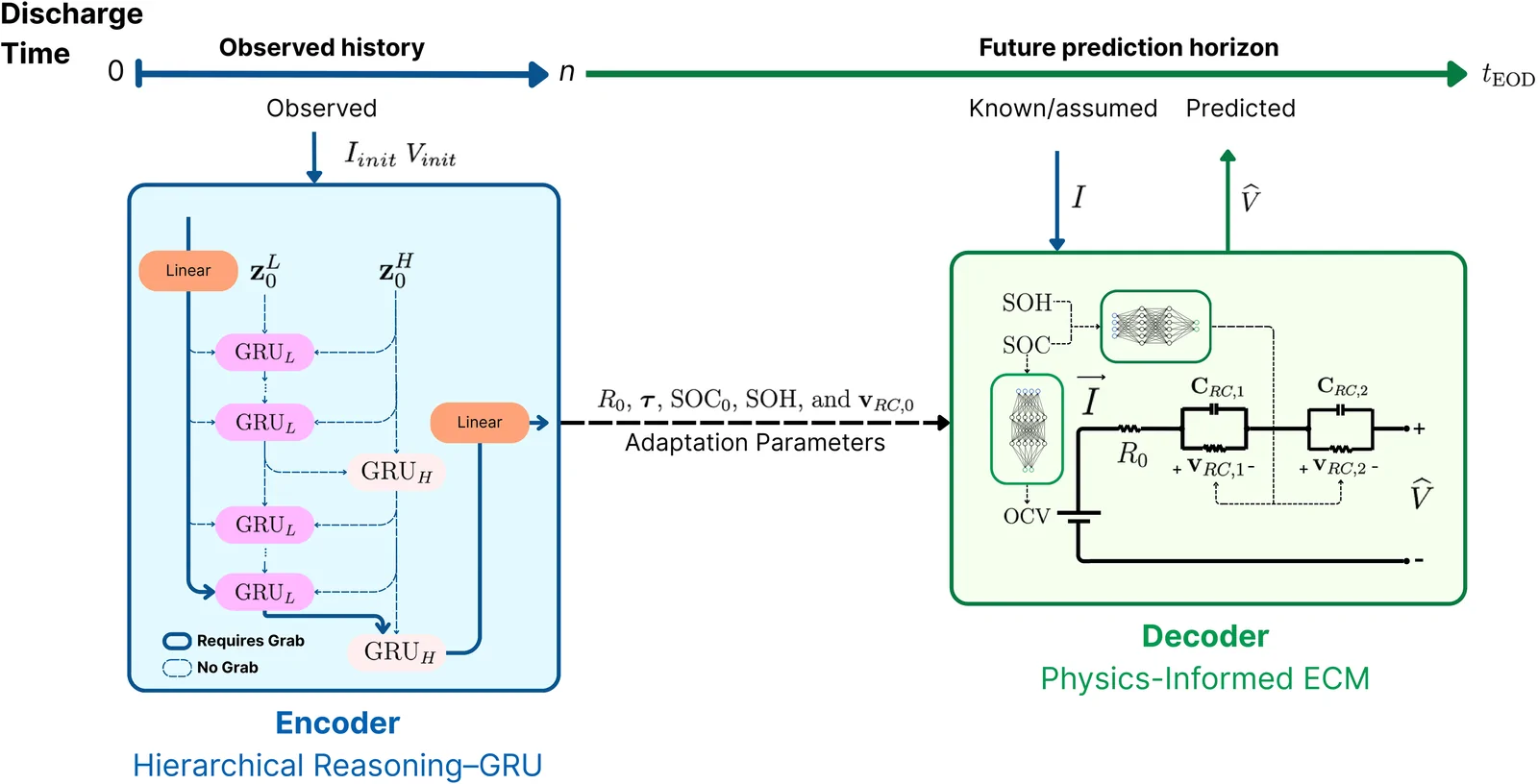
Overall architecture of our proposed SeqBattNet.

### 3.1 Encoder: Adaptation parameter prediction

Our encoder leverages the Hierarchical Reasoning Model (HRM) [39] to design a Hierarchical Reasoning–GRU encoder. HRM provides a multi-level reasoning framework that enables effective representation of complex temporal dependencies in the battery state during each cycle. Whereas [39] employs Transformers for hierarchical reasoning, we replace Transformers with GRUs. This substitution reduces computational complexity and improves prediction accuracy, as demonstrated in the experiment section. For each discharge cycle, the Hierarchical Reasoning–GRU encoder is provided with an initial segment of current and voltage, from the start of discharge up to a fixed length of *n* time steps, to estimate the battery adaptation parameters: the ohmic resistance *R*_0_ (capturing the instantaneous voltage drop), the RC branch time constants $\tau =\{\tau_{1},...,\tau_{e_{rc}}\}$ (describing diffusion and polarization dynamics), the initial state of charge SOC_0_, the state of health SOH (indicating capacity degradation at the current discharge cycle), and the initial RC branch voltages ${𝐯}_{RC,0}$. In this work, we set the number of RC branches to *e*_rc_ = 2. The detailed architecture of the Hierarchical Reasoning–GRU encoder block is presented as follows.

At each discharge cycle, the initial sequences of current $I_{init}\in {\mathbb{R}}^{n}$ and voltage $V_{init}\in {\mathbb{R}}^{n}$ are combined into $𝐱=[I_{init},V_{init}]\in {\mathbb{R}}^{n\times 2}$, which serves as the input to the Hierarchical Reasoning–GRU encoder. Here, *n* denotes the sequence length. The Hierarchical Reasoning–GRU first maps the input **x** into an embedding space through a linear layer:


$$\begin{array}{c} 𝐞=𝐱{𝐖}_{emb}+{𝐛}_{emb}\;\in \;{\mathbb{R}}^{n\times d_{emb}}, \end{array}$$ (2)

where *d*_emb_ denotes the embedding dimension. ${𝐖}_{emb}\in {\mathbb{R}}^{2\times d_{emb}}$ is the weight matrix of the linear layer, and ${𝐛}_{emb}\in {\mathbb{R}}^{d_{emb}}$ is the bias vector.

The core component of the hierarchical reasoning architecture, comprising the preparatory micro-update and main update phases, is used to process the embedding **e**. We begin with the preparatory micro-update phase. Let ${𝐳}_{s}^{L}\in {\mathbb{R}}^{d_{L}}$ and ${𝐳}_{s}^{H}\in {\mathbb{R}}^{d_{H}}$ denote the low- and high-level hidden states at sequence index s=0,...,n along the time dimension of the input **x**, with initial states ${𝐳}_{0}^{L}=0$ and ${𝐳}_{0}^{H}=0$. Here, $d_{L}$ and $d_{H}$ represent the dimensionalities of the low- and high-level latent spaces, respectively. Before consuming ${𝐞}_{s}$, we perform Q=NT−1 gradient-free micro-updates, where *N* denotes the number of reasoning layers and *T* denotes the number of update steps per layer, in order to obtain the pre-states $\left({𝐳}_{s^{-}}^{L},{𝐳}_{s^{-}}^{H}\right)$. We formalize this process through the gradient-free micro-update operator ${𝒰}_{N,T}(\cdot )$, defined as


$$\begin{array}{c} ({𝐳}_{s^{-}}^{L},{𝐳}_{s^{-}}^{H})={𝒰}_{N,T}\;({𝐳}_{s-1}^{L},{𝐳}_{s-1}^{H};\;{𝐞}_{s}), \end{array}$$ (3)

where Q=NT−1 denotes the total number of micro-updates. Specifically, for q=1,...,Q,


$$\begin{array}{cc} {𝐳}_{\left(q\right)}^{L} & ={GRU}_{L}\;([{𝐞}_{s},\;{𝐳}_{\left(q-1\right)}^{H}],\;{𝐳}_{\left(q-1\right)}^{L}),\\ {𝐳}_{\left(q\right)}^{H} & =\left\{ \begin{array}{cc} {GRU}_{H}\;({𝐳}_{\left(q\right)}^{L},\;{𝐳}_{\left(q-1\right)}^{H}), & \text{if }q\equiv 0\;(\mathrm{mod}\;T),\\ {𝐳}_{\left(q-1\right)}^{H}, & \text{otherwise}, \end{array}\right. \end{array}$$ (4)

with assignment


$$\begin{array}{c} ({𝐳}_{s^{-}}^{L},{𝐳}_{s^{-}}^{H})=({𝐳}_{\left(Q\right)}^{L},{𝐳}_{\left(Q\right)}^{H}). \end{array}$$ (5)

Where ${𝐳}_{s^{-}}^{L}$ and ${𝐳}_{s^{-}}^{H}$ denote the low- and high-level hidden states obtained after *Q* preparatory micro-updates.

In the main update phase, the current input embedding ${𝐞}_{s}$ is integrated into the hierarchical states:


$$\begin{array}{cc} {𝐳}_{s}^{L} & ={GRU}_{L}\;([{𝐞}_{s},\;{𝐳}_{s^{-}}^{H}],\;{𝐳}_{s^{-}}^{L}),\\ {𝐳}_{s}^{H} & ={GRU}_{H}\;({𝐳}_{s}^{L},\;{𝐳}_{s^{-}}^{H}). \end{array}$$ (6)

Here, ${𝐳}_{s}^{L}$ and ${𝐳}_{s}^{H}$ represent the updated hidden states after incorporating ${𝐞}_{s}$ through the low-to-high GRU updates. The notation [·,·] indicates feature concatenation. A dropout layer is then applied to ${𝐳}_{s}^{H}$ for prediction. After completing the high-level update at step *s*, we apply layer normalization to the hidden state ${𝐳}_{s}^{H}$, followed by a linear projection:


$$\begin{array}{c} {𝐨}_{s}\;=\;{𝐖}_{\text{out}}\;Norm({𝐳}_{s}^{H})+{𝐛}_{\text{out}}\;\in \;{\mathbb{R}}^{d_{\text{out}}}, \end{array}$$ (7)

where $d_{\text{out}}=2\;e_{rc}+3$, and Norm(·) denotes layer normalization [40]. Stacking across the *n* time steps yields $[{𝐨}_{1},...,{𝐨}_{n}]\in {\mathbb{R}}^{n\times d_{\text{out}}}$. We use the final vector as output, yielding:


$$\begin{array}{c} {}[R_{0},\;\tau ,\;{SOC}_{0},\;SOH,\;𝐰]={𝐨}_{n} \end{array}$$ (8)

where $R_{0}\in \mathbb{R}$, $\tau \in {\mathbb{R}}^{\;e_{rc}}$, ${SOC}_{0}\in \mathbb{R}$, SOH∈ℝ, and $𝐰\in {\mathbb{R}}^{\;e_{rc}}$. To ensure that these parameters remain physically valid, range-safe mappings are applied as follows:


$$\begin{array}{cc} R_{0} & \;\mapsto \;Aff\text{-}\sigma (R_{0};\;10^{-3},\;0.5),\\ \tau & \;\mapsto \;Aff\text{-}\sigma (\tau ;\;10^{-2},\;10^{5}),\\ {SOC}_{0} & \;\mapsto \;\sigma ({SOC}_{0}),\\ SOH & \;\mapsto \;\sigma (SOH),\\ 𝐰 & \;\mapsto \;Softmax(𝐰). \end{array}$$ (9)

where Aff-σ(z;ℓ,h)=ℓ+(h−ℓ)σ(z). Aff-σ is an affine-sigmoid mapping that constrains a parameter to a prespecified range [ℓ,h], σ is the standard sigmoid mapping to (0,1), and Softmax ensures normalized non-negative weights. The specific bounds (10^–3^, 0.5 for *R*_0_ and $10^{-2},10^{5}$ for τ) are chosen based on typical ranges of ohmic resistance [41] and RC time constants observed in lithium-ion cells [42], thereby enforcing physical plausibility. The vector ${𝐯}_{RC,0}\in {\mathbb{R}}^{\;e_{rc}}$, which provides the initial RC branch voltages, is computed as


$$\begin{array}{cc} {OCV}_{0} & \;=\;V_{EOD}+(V_{OCV,max}-V_{EOD})\;\sigma (g({SOC}_{0}))\\ s_{0} & \;=\;{OCV}_{0}\;-\;R_{0}I_{\text{last}}\;-\;V_{\text{last}},\\ {𝐯}_{RC,0} & \;=\;𝐰\;s_{0}\in {\mathbb{R}}^{\;e_{rc}}, \end{array}$$ (10)

where *I*_last_ and *V*_last_ denote the last observed current and voltage in the input window; $V_{OCV,max}$ and *V*_EOD_ represent the nominal voltage and the end-of-discharge cutoff voltage, respectively; OCV_0_ is the open-circuit voltage at time step *n* + 1; **w** denotes the weights associated with each RC branch; and g(·) is a feedforward neural network. Equation 10 will be explained in the next subsection 3.2.

The hyperparameters of Hierarchical Reasoning–GRU are summarized in Table 1. They are obtained using grid search [43]. The parameters *N* and *T* are searched within the range {1, 2, 3}. We restrict *N* and *T* to small values to limit the computational complexity of the hierarchical reasoning architecture. $d_{emb}$, $d_{L}$ and $d_{H}$ are searched over the set {16, 32, 64, 128, 256}. The operation of the decoder is presented in the next subsection.

**Table 1:** Hierarchical reasoning–GRU hyperparameters.

Parameter	Value
*N*	1
*T*	2
*d*_emb_	32
$d_{L}$	128
$d_{H}$	64

The resulting parameters *R*_0_, τ, SOC_0_, SOH, and ${𝐯}_{RC,0}$ are used as aging adaptation context and, together with the input current, are fed into the decoder to predict the terminal voltage.

### 3.2 Decoder: Terminal voltage prediction

The decoder implements a physics-informed neural network, inspired by the traditional first-order ECM [44], in which feedforward neural networks (FNNs) are employed to predict the open-circuit voltage and the resistances of the RC branches. The adaptation parameters predicted by the encoder block are then used to estimate the terminal voltage at each time step based on the input current.

**Algorithm 1. Decoder: Physics-Informed ECM Update for $m=n+1,...,t_{EOD}$.**

**Require:** Adaptation parameters: *R*_0_, τ, SOC_0_, SOH, ${𝐯}_{RC,0}$; battery parameters: *C*_rated_, *C*_EOL_, β; and current input sequence **I**.

1: Compute effective capacity: $C_{eff}=\beta C_{EOL}+(C_{rated}-\beta C_{EOL})SOH$

2: Compute decay coefficients: α=exp(−Δt/τ)

3: **for** *m* = *n* + 1 to *t*_EOD_ **do**

4: **OCV prediction:**


$$\begin{array}{c} {OCV}_{m}=V_{EOD}+(V_{OCV,max}-V_{EOD})\;\sigma (g({SOC}_{m})) \end{array}$$ (11)

5: **RC branch update:**


$$\begin{array}{cc} {𝐫}_{RC,m} & =r_{\min}+(r_{\max}-r_{\min})\;\sigma \;(f({SOC}_{m},SOH)),\\ {𝐯}_{RC,m} & =\alpha {𝐯}_{RC,m-1}+(1-\alpha )\;{𝐫}_{RC,m}I_{m}. \end{array}$$ (12)

6: **SOC update:**


$$\begin{array}{c} {SOC}_{m+1}=\Pi_{\left[0,1\right]}\;\left({SOC}_{m}-\frac{I_{m}\Delta t}{3600\;C_{eff}}\right) \end{array}$$ (13)

7: **Terminal voltage prediction:**


$$\begin{array}{c} {\hat{V}}_{m}={OCV}_{m}-R_{0}I_{m}-\sum_{k=1}^{e_{rc}}v_{RC,m,k} \end{array}$$ (14)

8: **end for**

**Ensure:** Predicted voltage sequence:


$$\begin{array}{c} \hat{V}=[V_{0}^{obs},...,V_{n}^{obs},\;{\hat{V}}_{n+1},...,{\hat{V}}_{t_{EOD}}] \end{array}$$ (15)

The overall algorithm of the decoder block is presented in Algorithm 1. The initial parameters from the encoder block, $R_{0}\in \mathbb{R}$, $\tau \in {\mathbb{R}}^{\;e_{rc}}$, ${SOC}_{0}\in \mathbb{R}$, SOH∈ℝ, and ${𝐯}_{RC,0}\in {\mathbb{R}}^{\;e_{rc}}$, together with the decay coefficient α and the current sequence $𝐈=\{I_{n+1},I_{n+2},...,I_{t_{EOD}}\}$, where each $I_{m}\in \mathbb{R}$ is positive for discharge and negative for charge, are used as inputs to the decoder to generate the terminal voltage at each time step $m=n+1,n+2,...,t_{EOD}$. Here, *t*_EOD_ denotes the step at which the battery reaches the EOD threshold. The decay coefficient α is defined as α=exp(−Δt/τ), which serves as a discrete-time decay factor that captures how the RC branch voltage relaxes over each sampling interval Δt. Both τ and Δt are expressed in seconds.

At each time step *m*, the open-circuit voltage $OCV_{m}\in \mathbb{R}$ is modeled by a feedforward neural network g(·) that maps the $SOC_{m}$ into the voltage range $\left[V_{EOD},V_{OCV,max}\right]$, as defined in Eq. 11. $V_{OCV,max}$ denotes the prescribed upper voltage bound of the learned OCV mapping, and *V*_EOD_ denotes the end-of-discharge cutoff voltage. The OCV represents the equilibrium voltage of the battery at no load, linking SOC to the intrinsic electrochemical potential.

For the RC branches, the resistance ${𝐫}_{RC,m}\in {\mathbb{R}}^{\;e_{rc}}$ is modeled by a feedforward neural network f(·,·) that takes ${SOC}_{m}$ and SOH as inputs and maps them into the range $\left[r_{min},\;r_{max}\right]$, as defined in Eq. 12. In our study, we set $r_{min}=10^{-4}$ and *r*_max_ = 1 as physically recommended constraints following [41]. The ${𝐫}_{RC,m}$ is used to compute the branch voltage ${𝐯}_{RC,m}$ at each time step. The ${𝐯}_{RC,m}\in {\mathbb{R}}^{\;e_{rc}}$ are calculated based on the decay coefficient α, the estimated ${𝐫}_{RC,m}$, and the current $I_{m}$. These voltages represent the dynamic relaxation behavior of the RC branches, capturing the delayed response of the battery to current excitation.

For the SOC update, ${SOC}_{m+1}\in \mathbb{R}$ is computed for the next time step *m* + 1, as defined in Eq. 13. Note that SOC_0_, predicted by the encoder, represents the initial SOC at the beginning of the prediction horizon (*m* = *n* + 1). The operator $\Pi_{\left[0,1\right]}$ denotes clipping to the interval [0,1].

The effective capacity *C*_eff_ is calculated from the encoder-predicted SOH, as specified in Algorithm 1, where *C*_rated_ denotes the nominal rated capacity and *C*_EOL_ denotes the end-of-life capacity, typically set to 0.8 *C*_rated_. Since the encoder processes an initial discharge segment before voltage prediction begins, we introduce a capacity scaling factor β=0.8, selected through grid search over {1.0, 0.9, 0.8, 0.7}.

The detailed architectures of g(·) and f(·) are presented in Table 2, with hyperparameters selected through grid search over {16, 32, 64, 128, 256}.

**Table 2:** FNN hyperparameters.

Network	Layer	Input	Output
g(·)	Linear+SiLU	1	32
	Linear+SiLU	32	32
	Linear	32	1
f(·)	Linear+SiLU	2	32
	Linear	32	*e*_rc_

For terminal voltage prediction, ${\hat{V}}_{m}\in \mathbb{R}$ is computed from the estimated $OCV_{m}$, the estimated ${𝐯}_{RC,m}$, the estimated *R*_0_, and the current $I_{m}$, as defined in Eq. 14. This ensures that the predicted terminal voltage accounts for both the instantaneous resistive drop across *R*_0_ and the dynamic relaxation behavior of the RC branches.

Finally, the predicted voltage sequence $\hat{𝐕}$ concatenates the observed historical voltages $V_{m}^{obs}$ (m=0,...,n) with the predicted future voltages ${\hat{V}}_{m}$ ($m=n+1,...,t_{EOD}$), as defined in Eq. 15. Here, $V_{m}^{obs}$ denotes the measured terminal voltage at time step *m*. The predicted terminal voltage is optimized using the proposed loss function, which is introduced in the next subsection.

### 3.3 Loss function

We propose a weighted loss function ${ℒ}_{wL1}$ based on the Huber loss [45] $\ell_{\delta_{Huber}}$ with adaptive weighting across the sequence. This formulation combines the robustness of the $\ell_{1}$ loss against outliers with the stability of the $\ell_{2}$ loss near zero error, while additionally emphasizing more critical time steps within the sequence. For a sequence of length *t*_EOD_, the loss is defined as


$$\begin{array}{c} {ℒ}_{wL1}=\frac{1}{\sum_{i=1}^{t_{EOD}}a_{i}w_{i}}\sum_{i=1}^{t_{EOD}}a_{i}\;w_{i}\;\ell_{\delta_{Huber}}\;\left({\hat{V}}_{i},V_{i}\right), \end{array}$$ (16)

where $V_{i}$ and ${\hat{V}}_{i}$ denote the true and predicted terminal voltages at time step *i*, $a_{i}\in \{0,1\}$ is a masking indicator, and $w_{i}$ is the adaptive weight. In the training process, all sequences are zero-padded to a common length, and a mask *a* is applied to restrict computations to the original valid entries. This procedure allows the model to be trained on batches of samples simultaneously rather than processing individual sequences sequentially.

The Huber loss $\ell_{\delta_{Huber}}$ is given by


$$\begin{array}{c} \ell_{\delta_{Huber}}({\hat{V}}_{i},V_{i})=\left\{ \begin{array}{cc} \frac{1}{2\delta_{Huber}}(V_{i}-{\hat{V}}_{i}{)}^{2}, & |V_{i}-{\hat{V}}_{i}|<\delta_{Huber},\\ |V_{i}-{\hat{V}}_{i}|-\frac{1}{2}\delta_{Huber}, & \text{otherwise}, \end{array}\right. \end{array}$$ (17)

with $\delta_{Huber}=0.1$ in our implementation.

The weight sequence $\left\{w_{1},w_{2},...,w_{t_{EOD}}\right\}$ is designed to emphasize earlier prediction steps and the last prediction step. Specifically, for a subsequence of length *t*_EOD_, the weights are construc*t*ed as


$$\begin{array}{c} w_{i}=\frac{1}{2}(\underset{\text{linear schedule from 10 to 1}}{\underbrace{\left(10-\frac{9}{t_{EOD}-1}(i-1)\right)}}+\delta_{i,t_{EOD}}\cdot 30),\;i=1,...,t_{EOD}, \end{array}$$ (18)

where the first term decreases linearly from 10 at *i* = 1–1 at *i* = *t*_EOD_, and $\delta_{i,t_{EOD}}$ is an indicator that adds a large weight (30) to the last prediction step. The values 10 and 30 were chosen empirically after testing several candidates {5,10,20,30,40}. By emphasizing both the beginning and the end of the sequence, this weighting scheme guides the model to follow the overall discharge profile more accurately.

### 3.4 Evaluation metrics

To evaluate battery voltage prediction performance, we employ three commonly used metrics: Root Mean Square Error (RMSE), Mean Absolute Error (MAE), and Mean Absolute Percentage Error (MAPE), following [21]. These metrics are defined as follows:


$$\begin{array}{c} RMSE(𝐕,\hat{𝐕})=\sqrt{\frac{1}{t_{EOD}}\sum_{i=1}^{t_{EOD}}(V_{i}-{\hat{V}}_{i}{)}^{2}}, \end{array}$$ (19)

$$\begin{array}{c} MAE(𝐕,\hat{𝐕})=\frac{1}{t_{EOD}}\sum_{i=1}^{t_{EOD}}|V_{i}-{\hat{V}}_{i}|, \end{array}$$ (20)

$$\begin{array}{c} MAPE(𝐕,\hat{𝐕})=\frac{1}{t_{EOD}}\sum_{i=1}^{t_{EOD}}\frac{\left|V_{i}-{\hat{V}}_{i}\right|}{\left|V_{i}\right|}. \end{array}$$ (21)

Here, $V_{i}$ denotes the measured terminal voltage at time step *i*, and ${\hat{V}}_{i}$ denotes the corresponding predicted voltage. Lower values of RMSE, MAE, and MAPE indicate better predictive performance. MAPE is reported as a dimensionless fraction rather than a percentage, consistent with its implementation in scikit-learn.

As this study focuses on battery voltage modeling, evaluation is limited to voltage prediction accuracy; EOD timing errors are not assessed.

## 4 Dataset

**TRI Dataset [**12**] – Identical Discharge Profiles for All cells.** The dataset includes 124 LFP/graphite A123 APR18650M1A LIBs, each with a nominal capacity of 1.1 Ah and a nominal voltage of 3.6 V. While various charge protocols are applied, all cells share identical discharge load profiles across cycles, ensuring highly consistent operating conditions. Cycle lives span from 150 to 2,300 cycles. Cells were discharged at a constant current of 4C down to 2 V, followed by a constant-voltage phase at 2 V until the current declined to C/50. Charging was conducted at rates between 3.6C and 6C under a controlled temperature of 30 ° C. With approximately 96,700 recorded cycles, this is among the largest publicly available datasets for studying fast-charging behavior. In this dataset, cell b1c0 is used for training, b2c13 for validation, and cells b1c4 and b1c2 are reserved for testing. Accordingly, we set the model parameters to *C*_rated_ = 1.1 Ah, *C*_EOL_ = 0.88 Ah (i.e., 0.8 *C*_rated_), *V*_OCV,max_ = 3.6 V, and *V*_EOD_ = 2.0 V. The dataset is publicly available at https://data.matr.io/1/.

**RT-Batt Dataset [**46**] – Different Discharge Profiles for cells.** The dataset consists of 77 LFP/graphite A123 APR18650M1A cells (1.1 Ah, 3.6 V nominal OCV), each subjected to a unique multi-stage discharge profile that remains consistent across cycles. All cells followed an identical fast-charging protocol and were tested in thermostatic chambers at 30 ° C. The dataset includes 146,122 discharge cycles, making it one of the largest for studying varied discharge behaviors. Cycle life ranges from 1,100 to 2,700 cycles. In this dataset, cell 1–1 is used for training, 1–4 for validation, and cells 1–2 and 1–3 are used for testing. Accordingly, we set the model parameters to *C*_rated_ = 1.1 Ah, *C*_EOL_ = 0.88 Ah (i.e., 0.8 *C*_rated_), *V*_OCV,max_ = 3.6 V, and *V*_EOD_ = 2.0 V. The dataset is publicly available at https://data.mendeley.com/datasets/nsc7hnsg4s/2

**NASA Dataset [**47**] – Different Discharge Profiles for both cells and cycles.** This dataset features high variability in discharge profiles across different cells and cycles, closely simulating real-world battery usage. Provided by the NASA Ames Prognostics Data Repository, it includes four 18650 lithium-ion cells (RW3–RW6), each charged at a constant 2 A to 4.2 V and discharged to 3.2 V using randomized current profiles between 0.5 A and 4 A. This variability makes the dataset ideal for testing model robustness under diverse operating conditions. In this dataset, cell RW3 is used for training, RW6 for validation, and cells RW4 and RW5 are used for testing. Accordingly, we set the model parameters to *C*_rated_ = 2.22 Ah, *C*_EOL_ = 1.33 Ah (i.e., 0.6 *C*_rated_), *V*_OCV,max_ = 4.2 V, and *V*_EOD_ = 3.2 V. The dataset is publicly available at https://github.com/wang-fujin/BattNN/tree/main/data/NASA11.

Table 3 summarizes the key characteristics of the selected datasets used in this study. The model is trained using data from a single cell and evaluated on unseen cells. This setting reflects a practical deployment scenario in which extensive labeled degradation data are unavailable for every battery. Using independent cells for validation and testing ensures that performance is assessed based on the model’s ability to generalize across different degradation trajectories rather than memorizing cell-specific patterns. Following prior battery prognostics studies, two cells are reserved for testing to provide a more reliable evaluation under unseen operating histories.

**Table 3:** Summary statistics and data split configuration of the datasets.

Dataset	Split	Cell(s)	No. of Cycles	Used Cells	Capacity (Ah)	*V*_*O C V*, m a x_ (V)	*V*_EOD_ (V)
TRI	Train	b1c0	1851	4	1.10	3.6	2.0
	Val	b2c13	520				
	Test	b1c4, b1c2	3944				
RT-Batt	Train	1−1	1504	4	1.10	3.6	2.0
	Val	1−4	1500				
	Test	1-2, 1-3	4536				
NASA	Train	RW3	100	4	2.22	4.2	3.2
	Val	RW6	100				
	Test	RW4, RW5	200				

Figure 2 provides a visualization of the discharge process across several cycles, from the beginning of life to the end of life, for three datasets. As batteries age, the voltage tends to decline more rapidly toward the end of discharge, which is particularly evident in the TRI and RT-Batt datasets. Each dataset exhibits different characteristics: the TRI dataset has identical discharge load profiles for cells and cycles; the RT-Batt dataset has different profiles for cells but identical profiles for cycles; and the NASA dataset has different profiles across both cells and cycles. These variations simulate different usage behaviors in real-world scenarios. Such behavior highlights the need to develop models that can adapt to both battery aging and varying load conditions.

**Fig 2:**
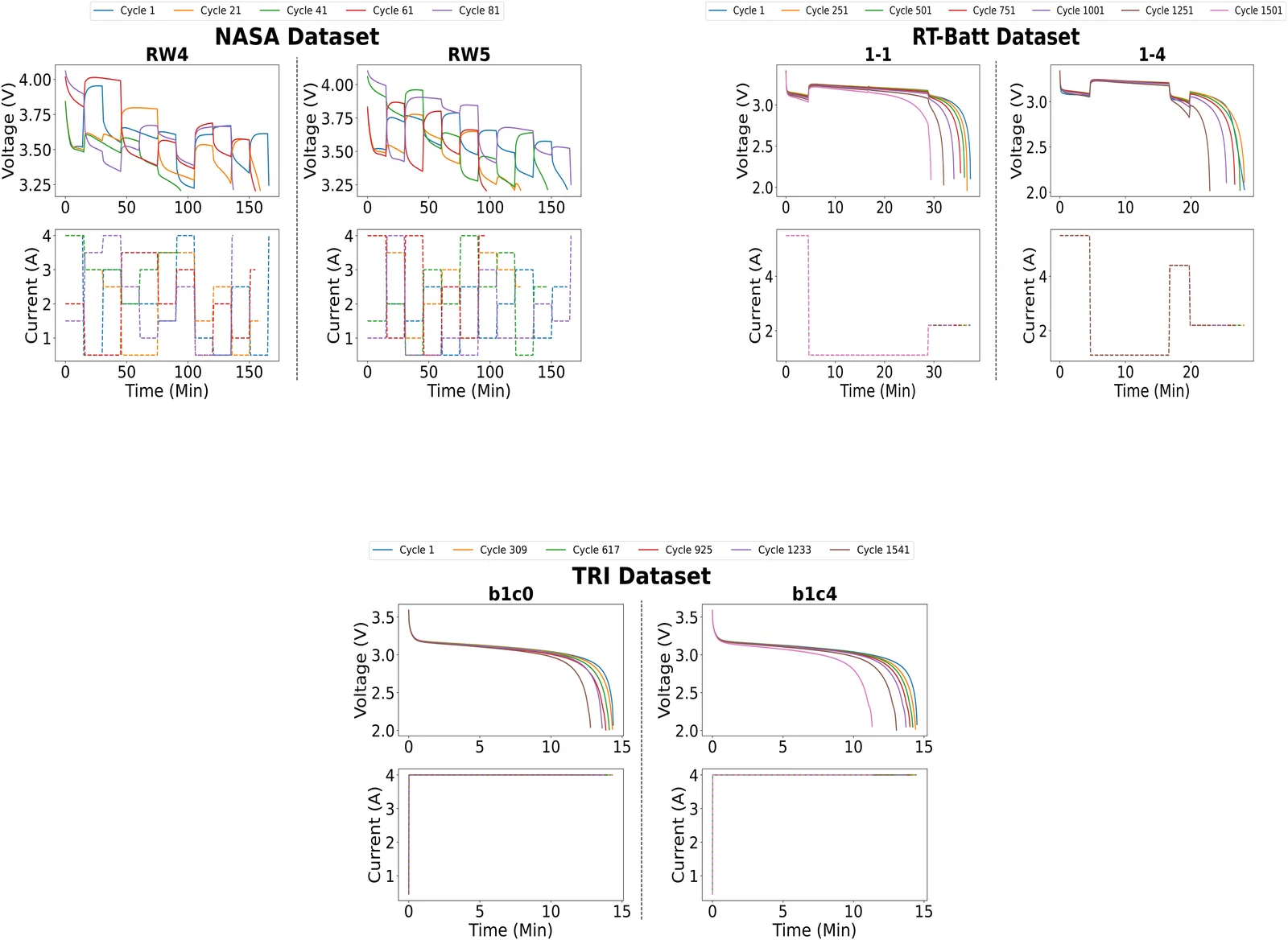
Visualization of the battery datasets: NASA, RT-Batt, and TRI.

Figure 3 presents the correlation heatmaps of the input variables across the three battery datasets. The heatmaps reveal distinct relationships between current, voltage, and temperature under different operating conditions. In the NASA dataset, current and voltage exhibit a moderate negative correlation (r=−0.43), reflecting the highly variable discharge profiles across cells and cycles. In contrast, the RT-Batt dataset shows a stronger positive correlation between current and voltage (*r* = 0.62), indicating more consistent discharge behavior under fixed discharge profiles. For the TRI dataset, current and voltage present a weak positive correlation (*r* = 0.29), while temperature demonstrates a relatively strong negative correlation with voltage (r=−0.67), suggesting that thermal effects may influence voltage behavior during battery operation. Overall, the correlation patterns highlight the diversity of operating conditions across datasets and support the need to evaluate the proposed model under highly variable scenarios, as demonstrated in the next experimental section.

**Fig 3:**
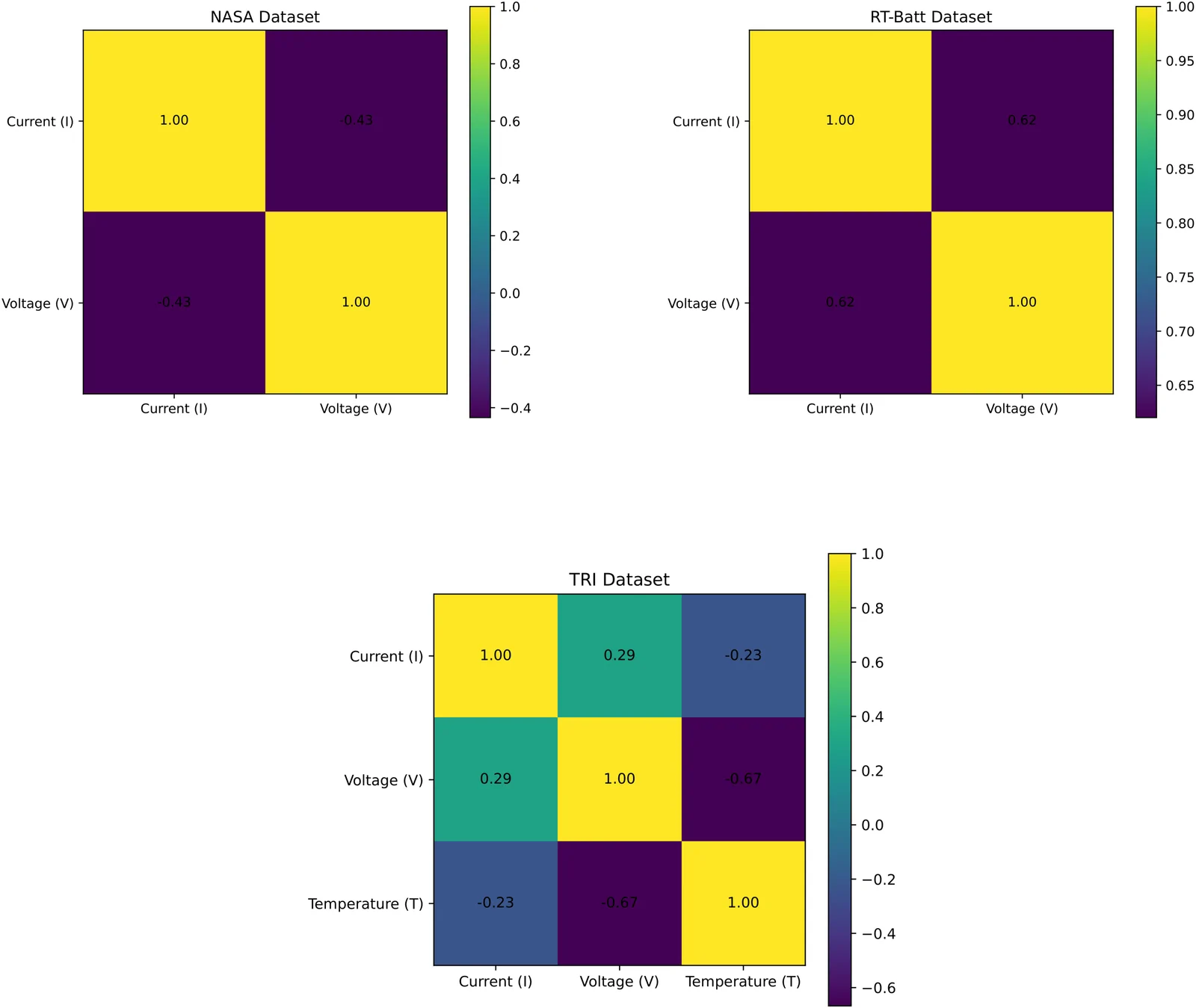
Correlation heatmaps of the battery datasets: NASA, RT-Batt, and TRI.

## 5 Experiment

### 5.1 Experimental setup

Our proposed SeqBattNet is implemented using the PyTorch framework. The model is trained using the AdamW optimizer [48]. All experiments are conducted on an NVIDIA RTX 4080 GPU with 16 GB of memory. Each model is trained for 1000 epochs with a batch size of 128. We employ PyTorch’s ReduceLROnPlateau scheduler with an initial learning rate of $2\times 10^{-3}$ (mode = min). If the validation loss does not improve for five consecutive epochs, the learning rate is multiplied by 0.8, with a minimum learning rate of $1\times 10^{-4}$.

We use the default random-number initialization in NumPy and PyTorch without manually fixing the random seeds. To reduce the influence of random initialization and training variability, each experiment is independently repeated five times. The predictions are averaged across runs before calculating RMSE, MAE, and MAPE for each test sample. The mean values across all test samples are reported in Tables 4–6.

For all datasets, only the discharge phase is used for battery modeling. Specifically, the initial segment of the discharge voltage and current signals is used as the encoder input, where the segment length is 80 samples for the TRI and RT-Batt datasets and 30 samples for the NASA dataset, as determined by the ablation study in Fig 4. These input signals are fed directly into the encoder without normalization. The encoder estimates the aging-adaptation parameters, which are subsequently provided to the decoder together with the current signal to predict the battery voltage. We do not normalize the input voltage and current because the initial linear layer serves as a learnable feature transformation, effectively learning an appropriate input representation during training.

**Fig 4:**
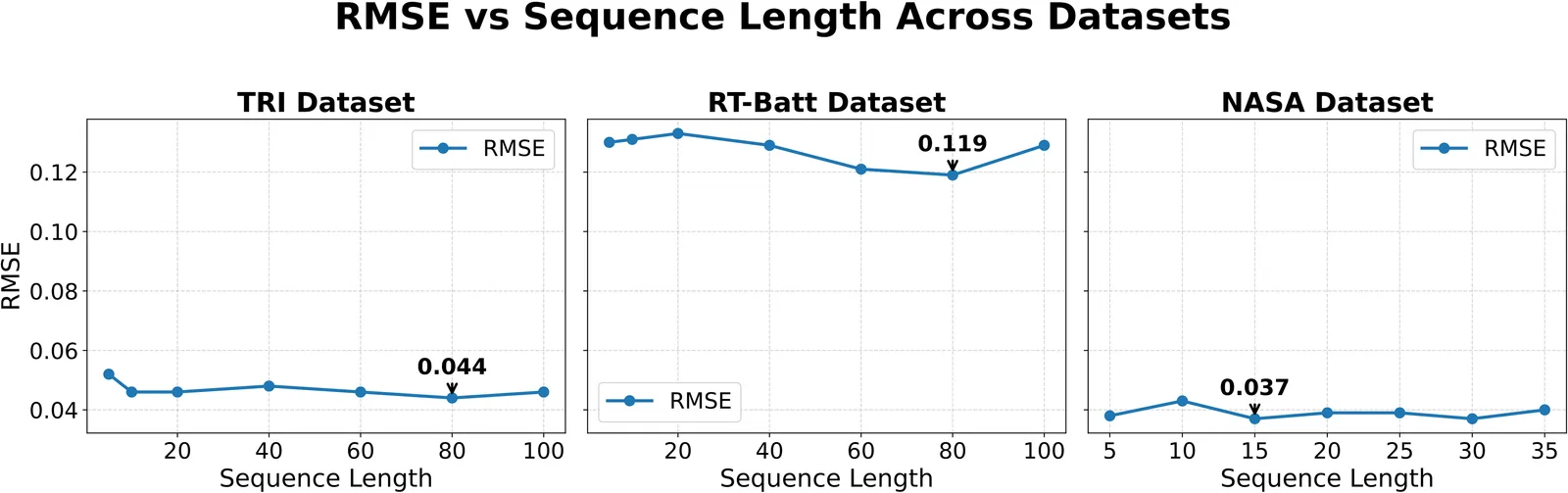
Impact of the initial sequence length used for aging adaptation on TRI, RT-Batt, NASA dataset.

The results of BattNN [21] and PDB-Net [49] are taken directly from their original papers. The LSTM, GRU, FNN, and Transformer baselines are implemented by us under the same experimental setup as SeqBattNet. Unless otherwise specified, these baseline models use a hidden dimension of 128, a single hidden layer, and the default PyTorch hyperparameter settings.

### 5.2 Effect of initial sequence length

The length of the initial sequence data **x** in each discharge cycle, used for aging adaptation, affects the prediction of aging adaptation parameters. We do not evaluate excessively long sequence lengths for aging adaptation within each cycle, since in practice users need to monitor battery states as early as possible. As shown in Fig 4, the TRI and RT-Batt datasets achieve their best performance with a sequence length of 80, while the NASA dataset performs best with a sequence length of 30. One of the reasons the TRI and RT-Batt datasets require a common initial segment length is that they use the same battery cell type A123 APR18650M1A. Each battery type, however, requires a different effective initial discharge length for aging detection. These sequence lengths are employed in the subsequent experiments.

### 5.3 Aging adaptation and loss function ablation study

Figure 5 illustrates the effect of the encoder on aging adaptation by projecting the learned embeddings, obtained from the encoder’s final layer, onto two principal components using principal component analysis (PCA) [50]. The trajectories show a clear progression with respect to the cycle index, where earlier cycles (purple/blue) gradually diverge from later cycles (green/yellow). This smooth transition indicates that the encoder effectively captures the temporal evolution of battery degradation. The clustering and gradual shifts across principal components suggest that our model is sensitive to aging dynamics, enabling it to distinguish between early-life and late-life behaviors.

**Fig 5:**
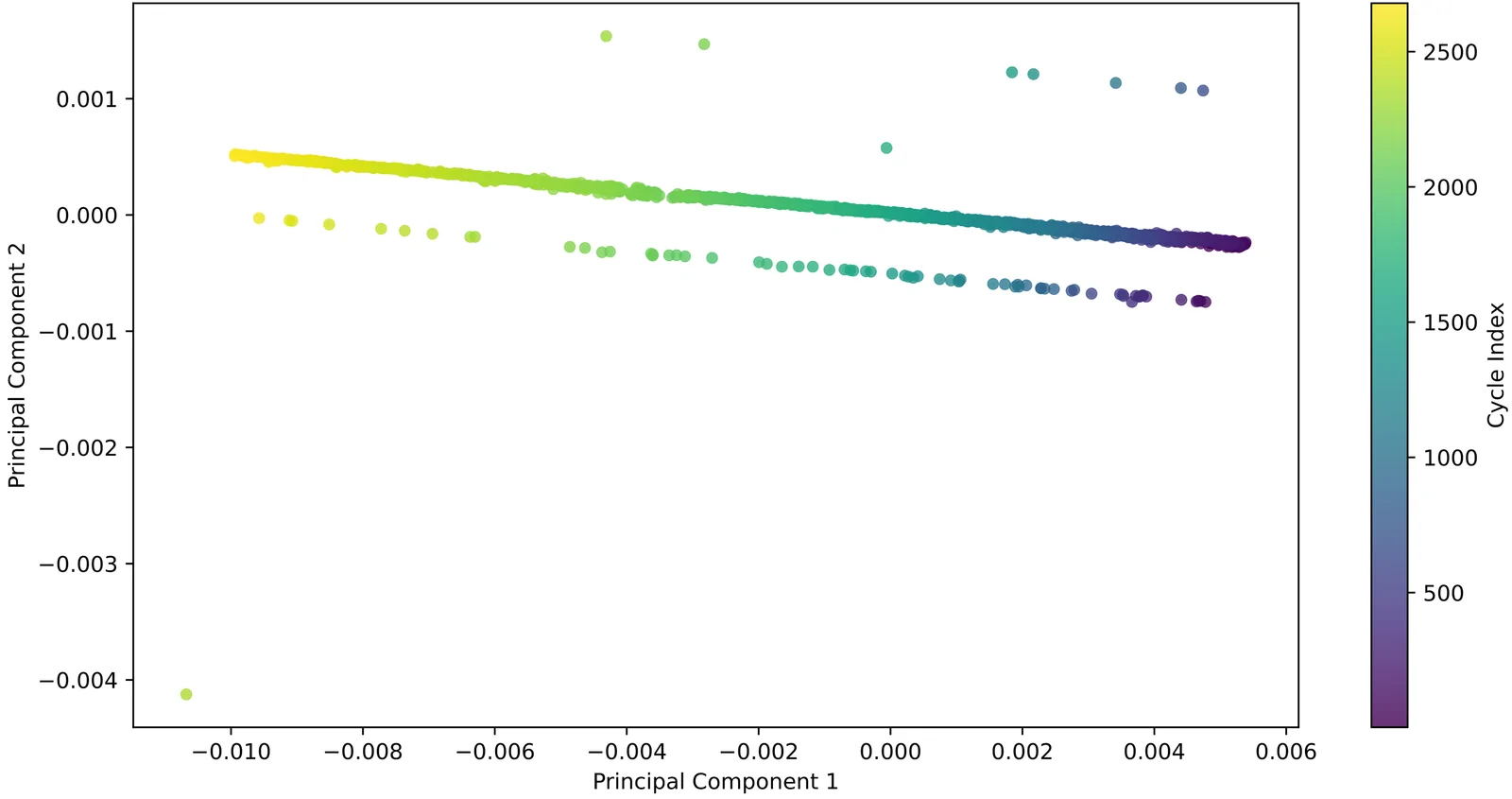
Aging trajectories visualized using PCA on the Encoder embeddings for Cell 1-2 of the RT-Batt Dataset.

Figure 6 illustrates the relative contribution of the encoder-generated physical parameters to the voltage prediction, quantified using the parameter ablation importance method [51]. Specifically, the importance of each parameter is measured by the mean absolute change in the predicted voltage trajectory after replacing that parameter with its dataset-average value while keeping the remaining parameters unchanged. The RC time constants (τ) are the most influential factors, accounting for 63.33% of the total importance, followed by the SOH-dependent effective capacity (29.62%). Together, these two aging-related parameters contribute more than 92% of the overall influence on the predicted voltage, indicating that the proposed encoder primarily adapts battery dynamics by learning the evolution of polarization behavior and available capacity throughout the aging process. In contrast, the contributions of the ohmic resistance (*R*_0_), the initial RC voltage, and the initial state of charge (SOC_0_) are comparatively small, accounting for only 2.75%, 3.82%, and 0.48%, respectively. These findings indicate that the encoder-generated parameters influence voltage prediction to varying degrees. However, their physical accuracy and unique identifiability have not been independently validated.

**Fig 6:**
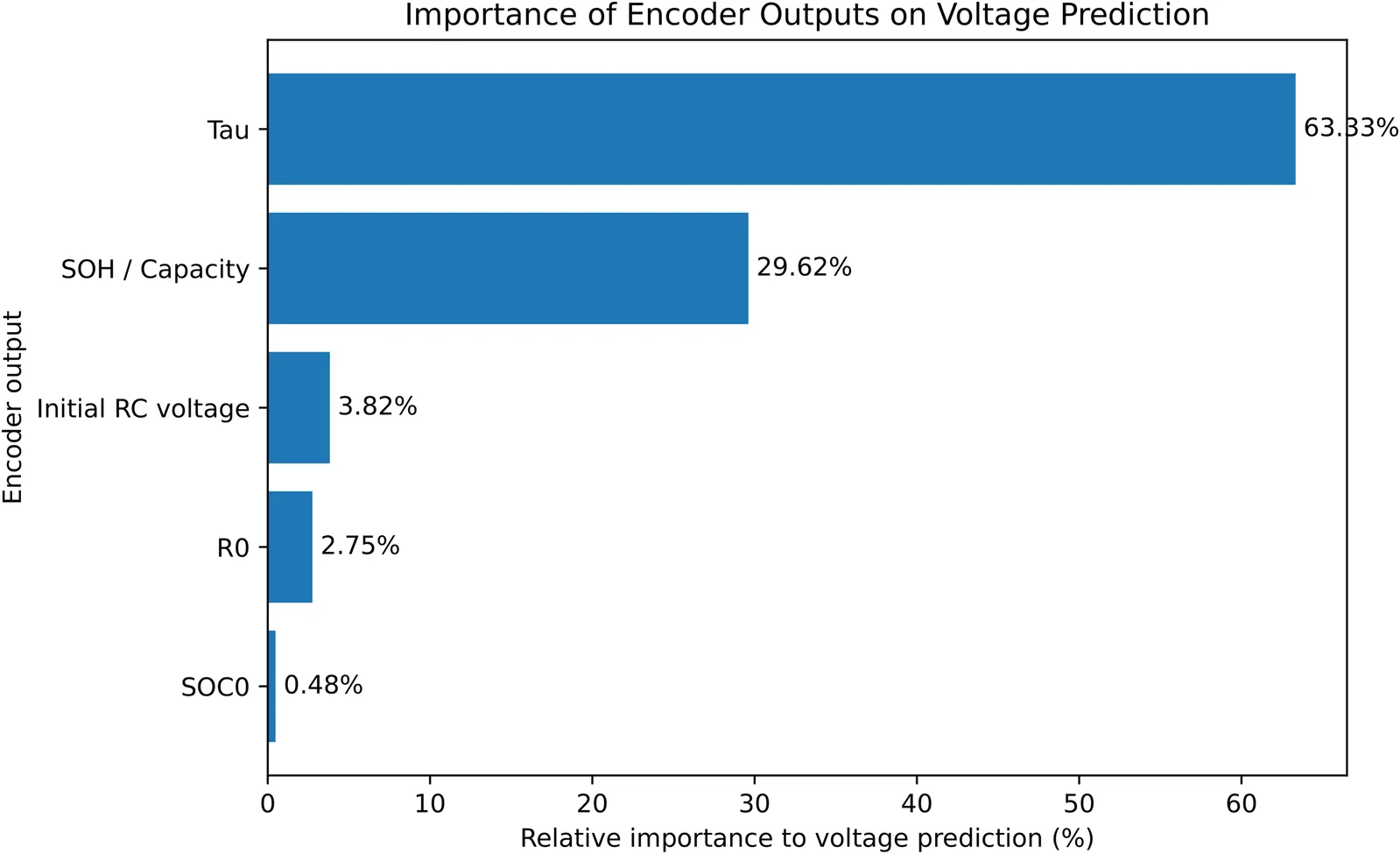
Relative importance of the encoder-generated physical parameters to voltage prediction.

Table 4 compares the proposed loss with MSE [52] and BattNN loss [21], with and without aging adaptation. With adaptation, the proposed loss achieves the lowest RMSE, MAE, and MAPE across all batteries. For example, RMSE decreases from 0.1299 (MSE) and 0.0624 (BattNN) to 0.0280 on TRI-b1c4, and from 0.0902 and 0.0579 to 0.0346 on RT-Batt-1–2. On NASA-RW4 and RW5, RMSE reaches 0.0257 and 0.0253.

**Table 4:** Comparison of the proposed loss function with baseline methods, with and without aging adaptation.

Dataset	Battery	With Aging Adaptation									Without Aging Adaptation								
		MSE			BattNN [21]			Ours			MSE			BattNN [21]			Ours		
		RMSE	MAE	MAPE	RMSE	MAE	MAPE	RMSE	MAE	MAPE	RMSE	MAE	MAPE	RMSE	MAE	MAPE	RMSE	MAE	MAPE
TRI	b1c4	0.1299	0.1003	0.0345	0.0624	0.0252	0.0096	0.0280	0.0113	0.0042	1.0501	0.9222	0.3089	0.0855	0.0369	0.0140	0.0747	0.0398	0.0147
	b1c2	0.1339	0.1038	0.0355	0.0680	0.0261	0.0099	0.0416	0.0168	0.0063	1.0606	0.9314	0.3108	0.0802	0.0317	0.0121	0.0705	0.0335	0.0124
RT-Batt	1-2	0.0902	0.0445	0.0155	0.0579	0.0196	0.0073	0.0346	0.0120	0.0044	0.0950	0.0503	0.0174	0.0841	0.0393	0.0139	0.1088	0.0460	0.0164
	1-3	0.0965	0.0499	0.0172	0.0593	0.0199	0.0074	0.0491	0.0171	0.0063	0.1004	0.0547	0.0188	0.0767	0.0372	0.0131	0.1002	0.0426	0.0153
NASA	RW4	0.0319	0.0226	0.0064	0.0288	0.0194	0.0056	0.0257	0.0191	0.0055	0.5471	0.3966	0.1095	0.0415	0.0319	0.0090	0.0392	0.0297	0.0084
	RW5	0.0329	0.0238	0.0067	0.0301	0.0204	0.0059	0.0253	0.0191	0.0055	0.5384	0.3884	0.1071	0.0429	0.0330	0.0094	0.0395	0.0297	0.0084

Without aging adaptation, encoder-generated parameters are replaced with fixed values selected through grid search on initial discharge cycles. For TRI and RT-Batt, which share the same cell type, the parameters are *R*_0_ = 0.05, $\tau =(4\times 10^{4},10^{4})$, SOH = 0.95, SOC_0_ = 1, and ${𝐯}_{RC,0}=(0.3,4\times 10^{-6})$. For NASA, they are *R*_0_ = 0.1, $\tau =(3\times 10^{4},2\times 10^{4})$, SOH = 0.95, SOC_0_ = 1, and **v**_RC,0_ = (0.2, 0.2). The decoder architecture, training data, and optimization settings remain unchanged within each loss-function comparison.

Without adaptation, RMSE increases from 0.0280 to 0.0747 on TRI-b1c4, 0.0346 to 0.1088 on RT-Batt-1–2, and 0.0253 to 0.0395 on NASA-RW5. These results support the benefit of cycle-specific adaptation, although differences in parameter fitting prevent its contribution from being fully isolated.

### 5.4 Encoder architecture ablation study

Table 5 presents an ablation study of different encoder architectures for SeqBattNet across the TRI, RT-Batt, and NASA datasets. The first block reports the performance of conventional sequence-learning baselines, including LSTM, GRU, and Transformer. A standalone FNN was not included as a conventional encoder baseline because its limited temporal modeling capacity makes it unsuitable for independently capturing sequential battery dynamics. Therefore, the FNN was evaluated only as an encoder component within SeqBattNet, where the physics-informed decoder provides additional structural guidance. The second block evaluates SeqBattNet using standard LSTM, GRU, Transformer, and FNN encoders, while the third block incorporates the HRM with the same encoder families.

**Table 5:** Ablation study of different encoder architectures for SeqBattNet on the TRI, RT-Batt, and NASA datasets.

Dataset	Battery	LSTM				GRU				Transformer							
		RMSE	MAE	MAPE	Time (s)	RMSE	MAE	MAPE	Time (s)	RMSE	MAE	MAPE	Time (s)				
TRI	b1c4	0.0712	0.034	0.0127	10.0834	0.0713	0.034	0.0127	9.4537	0.0762	0.0357	0.0133	32.6908				
	b1c2	0.0707	0.0327	0.0121	12.3596	0.0704	0.0314	0.0117	12.2257	0.0744	0.0323	0.0121	41.6712				
RT-Batt	1-2	0.1303	0.0516	0.0189	9.5352	0.2197	0.1027	0.0353	10.1433	0.145	0.0699	0.025	20.3147				
	1-3	0.134	0.0518	0.0191	6.3358	0.1114	0.0498	0.0177	6.3457	0.1513	0.0741	0.0265	12.944				
NASA	RW4	0.0297	0.0208	0.0059	0.1441	0.0272	0.0203	0.0058	0.1446	0.0251	0.017	0.0049	0.1884				
	RW5	0.0308	0.022	0.0062	0.1712	0.0281	0.021	0.0061	0.1618	0.027	0.0181	0.0052	0.1966				
**Dataset**	**Battery**	**SeqBattNet**				**SeqBattNet**				**SeqBattNet**				**SeqBattNet**			
		**LSTM for Encoder**				**GRU for Encoder**				**Transformer for Encoder**				**FNN for Encoder**			
		**RMSE**	**MAE**	**MAPE**	**Time (s)**	**RMSE**	**MAE**	**MAPE**	**Time (s)**	**RMSE**	**MAE**	**MAPE**	**Time (s)**	**RMSE**	**MAE**	**MAPE**	**Time (s)**
TRI	b1c4	0.0684	0.0284	0.0108	60.817	0.0742	0.0319	0.012	59.5293	0.0483	0.0211	0.0079	55.9238	0.0647	0.0268	0.0101	57.6439
	b1c2	0.0677	0.028	0.0105	78.6893	0.0736	0.0304	0.0114	76.7242	0.0528	0.0223	0.0083	73.3107	0.0653	0.0262	0.0099	73.64
RT-Batt	1-2	0.0827	0.032	0.0117	58.8327	0.0827	0.0346	0.0124	55.5968	0.0824	0.0323	0.0118	56.9902	0.0826	0.0319	0.0117	51.1914
	1-3	0.0723	0.0283	0.0103	38.4323	0.0845	0.0393	0.0139	36.5965	0.0726	0.0296	0.0108	37.1704	0.0721	0.0284	0.0104	33.3752
NASA	RW4	0.0259	0.0175	0.005	0.6694	0.0343	0.0234	0.0066	0.6745	0.0274	0.0181	0.0051	0.6676	0.0239	0.0155	0.0044	0.5531
	RW5	0.0264	0.0177	0.005	0.7327	0.0359	0.0249	0.0071	0.6741	0.0279	0.0183	0.0052	0.6768	0.0248	0.0159	0.0046	0.609
**Dataset**	**Battery**	**SeqBattNet**				**SeqBattNet**				**SeqBattNet**				**SeqBattNet**			
		**HRM-LSTM for Encoder**				**Hierarchical Reasoning–GRU for Encoder** ***(Proposed)***				**HRM-Transformer for Encoder**				**HRM-FNN for Encoder**			
		**RMSE**	**MAE**	**MAPE**	**Time (s)**	**RMSE**	**MAE**	**MAPE**	**Time (s)**	**RMSE**	**MAE**	**MAPE**	**Time (s)**	**RMSE**	**MAE**	**MAPE**	**Time (s)**
TRI	b1c4	0.0637	0.0255	0.0097	68.6676	0.028	0.0113	0.0042	68.819	0.0701	0.0298	0.0113	109.5333	0.0712	0.0317	0.0118	68.2895
	b1c2	0.0676	0.0265	0.0101	88.3893	0.0416	0.0168	0.0063	85.4199	0.0692	0.0291	0.0109	138.7852	0.0712	0.032	0.0118	87.8615
RT-Batt	1-2	0.0727	0.0264	0.0097	68.6494	0.0346	0.012	0.0044	70.2151	0.0827	0.032	0.0117	129.469	0.0813	0.0318	0.0116	72.7671
	1-3	0.0683	0.0324	0.0115	46.1185	0.0491	0.0171	0.0063	46.2879	0.0723	0.0286	0.0105	91.5932	0.0719	0.029	0.0106	46.3756
NASA	RW4	0.0307	0.0209	0.006	0.8571	0.0257	0.0191	0.0055	0.8675	0.029	0.0194	0.0055	1.7621	0.0315	0.0227	0.0065	0.87
	RW5	0.0313	0.0212	0.0061	0.9251	0.0253	0.0191	0.0055	0.8907	0.0293	0.0195	0.0055	1.7676	0.0325	0.0237	0.0068	0.9289

The results show that the proposed Hierarchical Reasoning–GRU encoder provides the strongest performance on the TRI and RT-Batt datasets. Specifically, it achieves RMSE values of 0.0280 and 0.0416 on TRI-b1c4 and TRI-b1c2, respectively, and 0.0346 and 0.0491 on RT-Batt-1–2 and RT-Batt-1–3, respectively. These results are substantially better than those obtained by the conventional sequence models, the standard SeqBattNet encoder variants, and the other HRM-based variants. This indicates that the hierarchical interaction between the low- and high-level GRU states effectively captures both short-term voltage dynamics and cycle-dependent battery degradation.

On the NASA dataset, the proposed Hierarchical Reasoning–GRU achieves RMSE values of 0.0257 on RW4 and 0.0253 on RW5. Although some simpler SeqBattNet variants, particularly the FNN encoder, obtain slightly lower RMSE values on this dataset, the proposed model maintains competitive prediction accuracy across all three datasets. This suggests that encoder effectiveness is dataset-dependent, while the Hierarchical Reasoning–GRU provides the most consistent overall performance under different discharge profiles and aging conditions. In terms of computational cost, the proposed model requires approximately 46–85 seconds on the TRI and RT-Batt datasets and less than one second on the NASA dataset.

### 5.5 Comparison with state-of-the-art methods

Table 6 compares the proposed method with BattNN and PDB-Net on the NASA dataset. The proposed method achieves the lowest RMSE, MAE, and MAPE on both RW4 and RW5. For RW4, it reduces the RMSE from 0.0504 with BattNN and 0.0382 with PDB-Net to 0.0257. Similarly, for RW5, the RMSE decreases from 0.0524 and 0.0406 to 0.0253, respectively. These results demonstrate that the proposed method provides more accurate and consistent voltage predictions than the existing physics-informed battery models on the evaluated NASA cells.

**Table 6:** Performance comparison of BattNN, PDB-Net, and the proposed method on the NASA dataset.

Dataset	Battery	BattNN [21]			PDB-Net [49]			Proposed		
		RMSE	MAE	MAPE	RMSE	MAE	MAPE	RMSE	MAE	MAPE
NASA	RW4	0.0504	0.0401	0.0113	0.0382	0.0283	0.0078	0.0257	0.0191	0.0055
	RW5	0.0524	0.0410	0.0116	0.0406	0.0311	0.0092	0.0253	0.0191	0.0055

Figure 7 illustrates the performance of our proposed SeqBattNet in predicting terminal voltage across three datasets: TRI, RT-Batt, and NASA. In each case, the predicted voltage trajectories (blue dashed lines) closely follow the actual measured voltages (black solid lines), demonstrating the model’s ability to capture both the overall discharge trend and local fluctuations. For the TRI dataset, the model accurately tracks the smooth discharge curve and the steep voltage drop near the end-of-discharge. For the RT-Batt dataset, SeqBattNet successfully captures the irregular step-like voltage behaviors caused by varying load conditions. For the NASA dataset, the model adapts to highly dynamic discharge profiles with sharp transitions and variable plateau regions. These results demonstrate that SeqBattNet achieves accurate terminal voltage predictions across the evaluated battery datasets under different discharge profiles.

**Fig 7:**
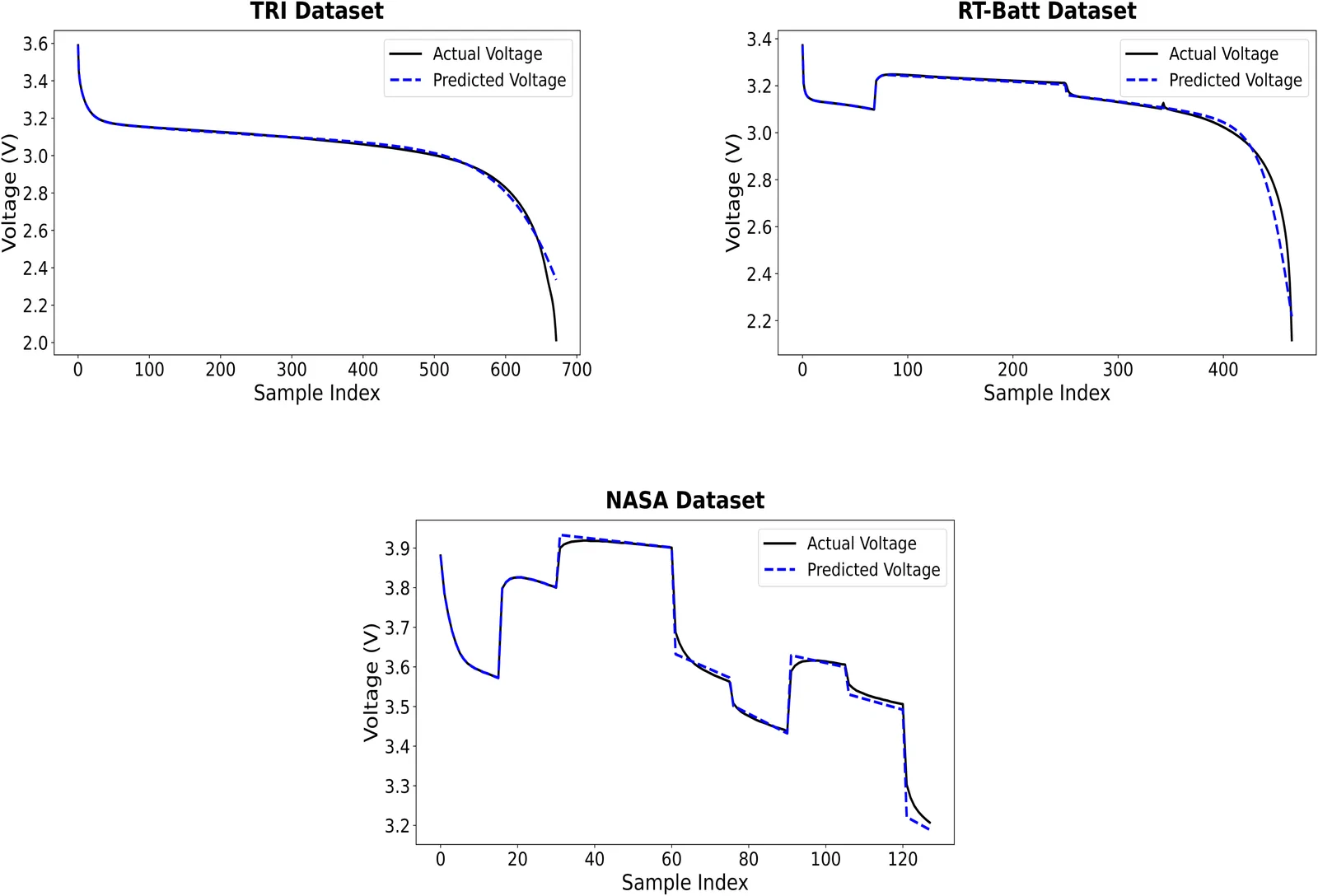
Visualization of terminal voltage prediction using our proposed SeqBattNet on the TRI, RT-Batt, and NASA datasets.

Figure 8 shows the mean RMSE and the corresponding 95% confidence intervals over five independent runs. The narrow confidence intervals across all batteries indicate that the proposed method achieves stable and reproducible performance. Although the prediction error varies among batteries, with RT-1-3 showing the highest RMSE and NASA-RW5 the lowest, the limited variability across runs demonstrates the robustness of the proposed method under different battery datasets.

**Fig 8:**
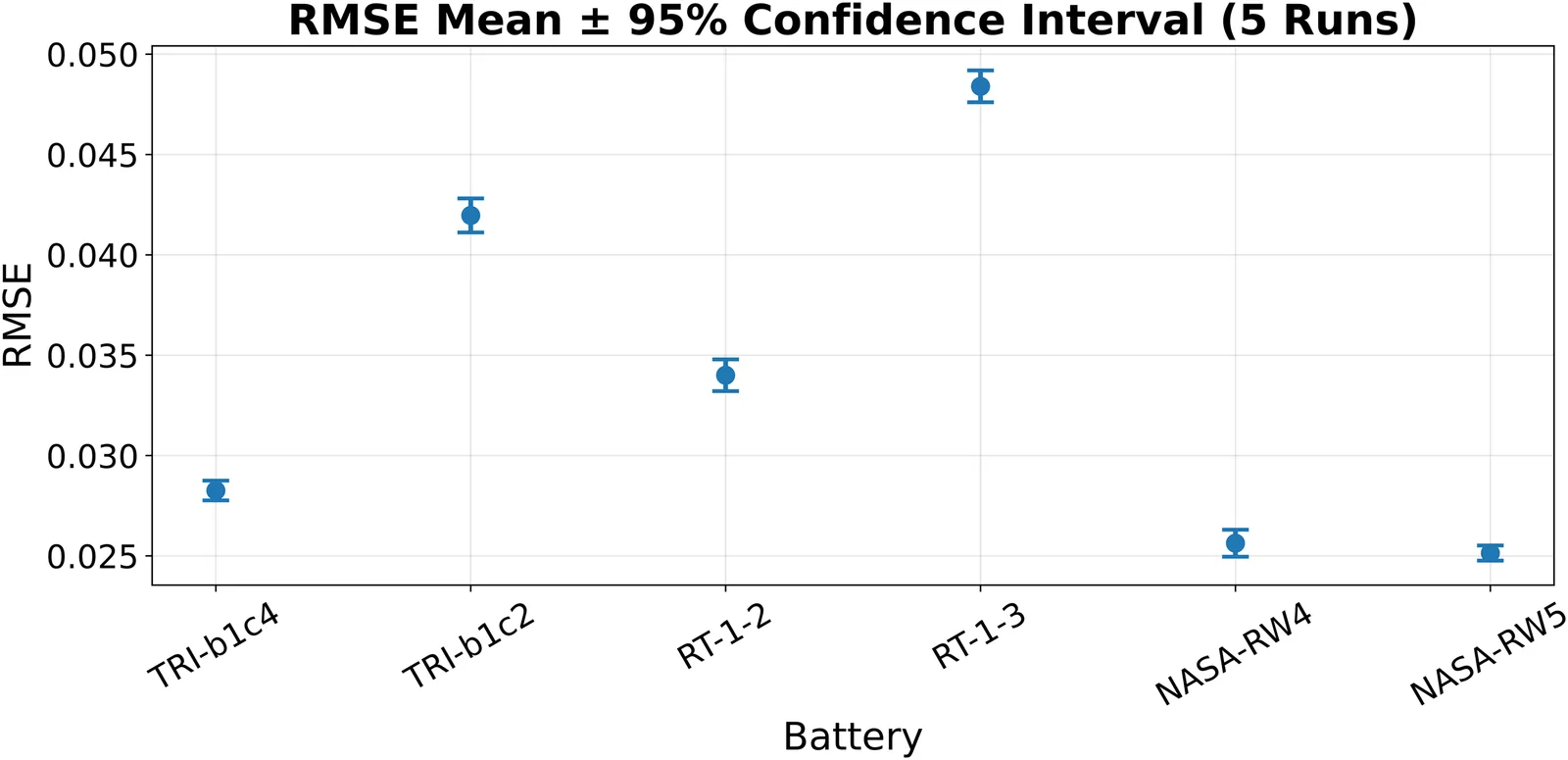
Mean RMSE values and 95% confidence intervals of the proposed method across five independent runs on different batteries from the TRI, RT-Batt, and NASA datasets.

### 5.6 Discussion and limitations

In this study, we focus solely on voltage and current as model inputs. Although we acknowledge that temperature strongly affects battery behavior, including capacity, internal resistance, degradation rate, and voltage response, temperature-related factors (e.g., ambient and cell temperature) are not considered in the current work. The main reason is that the public datasets employed were collected under controlled laboratory conditions, where temperature variations are minimal and do not fully represent real-world operating environments. In future work, we plan to extend the model by incorporating temperature features (both ambient and cell temperature) as additional inputs and evaluating the approach on real-world datasets that capture diverse environmental and operational conditions, thereby improving its generalizability beyond laboratory settings.

This study focuses exclusively on battery modeling, where terminal voltage is predicted under a given future current profile. Although EOD timing can be estimated from the predicted voltage trajectory, its accuracy is not evaluated in this work. Future work will extend the evaluation to EOD timing and RDT prediction.

## 6 Conclusion

This paper presented **SeqBattNet**, a discrete-state physics-informed neural network with built-in aging adaptation for battery voltage prediction during the discharge process. The proposed framework combines a Hierarchical Reasoning–GRU encoder that estimates cycle-specific physical parameters with an ECM-based decoder that performs interpretable voltage prediction. Extensive experiments on the TRI, RT-Batt, and NASA benchmark datasets demonstrate that SeqBattNet consistently outperforms both conventional sequence models and recent PINN-based approaches. In particular, on the NASA dataset, SeqBattNet achieves RMSE values of 0.0257 and 0.0253 for batteries RW4 and RW5, respectively, outperforming the recent PDB-Net by approximately 32.7% and 37.7%. The sensitivity analysis shows that the RC time constants and SOH-dependent effective capacity account for over 92% of the measured influence on voltage prediction. However, their physical accuracy and identifiability require further validation.

Although SeqBattNet demonstrates strong prediction accuracy and physical interpretability, several directions remain for future work. First, temperature information will be incorporated to better capture thermal effects on battery dynamics and aging. Second, the framework will be extended to jointly estimate battery states, such as SOC, SOH, and RUL. Finally, the proposed model will be validated on larger real-world datasets with diverse battery chemistries and operating conditions to further improve its robustness and practical applicability.

### Contact information

For access to the code and further information about this proposed system, please contact AIWARE Limited Company at: https://aiware.website/Contact.

### Preprint availability

A preprint of this manuscript is available at: https://arxiv.org/abs/2509.17621.
