# Supplementary material for: SeqBattNet: A discrete-state physics-informed neural network with aging adaptation for battery modeling
Source: PLoS One. 2026 Nov 12;21(11):e0362362. doi: 10.1371/journal.pone.0362362 (PMC13759123; doi:10.1371/journal.pone.0362362)
Supplement: Supplementary file 1 [file pone.0362362.s002.pdf]

Response Letter

We sincerely thank Editor-in-Chief, Associate Editor, and the reviewers for their constructive, pertinent, and useful comments on our manuscript.

To Editor-in-Chief and Associate Editor

We are pleased to see insightful and constructive comments from the reviewers. In this resubmission, we have attempted to address the reviewers' concerns and revised the manuscript accordingly. Further details about all the changes we made can be found in our point-to-point response to the reviewers' comments below. In the revised manuscript, we marked the changes in yellow.

To Reviewer 1

A background of different battery modelling should be presented as well as if physics informed models have been used previously.

Response: Thank you for your comment. We have expanded the Introduction section to provide additional background on battery modelling approaches. We have added a discussion of existing PINN-based battery modelling studies and clarified the motivation for using an ECM-based physics-informed formulation for RDT prediction.

Comment 2: All images are not clear and should be improved

Response: Thank you for your comment. Figure 1, which presents the overall architecture of the proposed SeqBattNet, has been improved to enhance clarity and readability.

Comment 3: section 4 on data set could do with a summary statistics and heat map. The section should also include a link to a publicly available data and code for the work.

Response: Thank you for your comment. We have added summary statistics and correlation heat maps for the three datasets. We have also included links to the public datasets. However, we cannot share the code for security reasons.

To Reviewer 2

Comment 1: The paper mainly contrasts SeqBattNet with a traditional sequence models (LSTM and GRU) and a small number of prior PINN-based methods. The review is weakened by the lack of comparisons with more recent state-of-the-art techniques because battery modeling and physics-informed machine learning are developing at a rapid pace. I recommend that you compare with more recent hybrid electrochemical-ML models or PINN-based models using relevant

publications from the past two to three years.

Response: Thank you for your comment. We have added more background information and included additional discussion of previous studies on PINNs in battery modeling in the Introduction section.

Comment 2: I understand that you mentioned that temperature effect is not considered in your work, I will suggest that you expand discussion on how this limitation affects real-world deployment. If possible, include preliminary analysis or justification for excluding temperature.

Response: Thank you for your comment. We have expanded the discussion in the Discussion and Limitations section to clarify the impact of excluding temperature effects on real-world deployment.

Comment 3: You highlighted data leakage in previous works, however, your experimental design raises concern regarding training diversity. The model is specifically trained on a single cell per dataset (Lines 278–279, 287–288, 296–297), which restricts the training data's variability and might not be adequate to support the asserted generalization across many cells. Additionally, neither the number of cycles used for training nor testing nor whether operating conditions (such as discharge profiles or deterioration phases) overlap between splits are explicitly mentioned in the study. Because of this lack of information, it is challenging to evaluate the evaluation's impartiality and robustness. It is recommended that the authors publish more detailed information on the training and testing splits and make clear how the dataset is used.

Response: Thank you for your comment. We added Table 3, which summarizes the dataset characteristics and provides detailed train/validation/test split configurations, including the selected cells and the number of cycles used in each split. In this study, we intentionally trained the model using data from a single battery cell and evaluated it on unseen cells for validation and testing to assess its ability to generalize across different degradation trajectories using only limited training data. This setup was chosen to reflect practical scenarios, where obtaining labeled battery degradation data is costly and time-consuming. This rationale has been clarified in the paragraph above Table 3.

Comment 4: The results reported in Tables 3 and 4 present only the mean performance metrics (RMSE, MAE, MAPE), despite the fact that each experiment is repeated multiple times (Lines 320–321). Reporting merely the mean could mask model performance variability since neural network training is stochastic. I urge that you provide the standard deviation (or confidence intervals) with the mean values in order to offer a more thorough and trustworthy assessment. This will assist in determining the stated improvements' stability and statistical significance.

Response: Thank you for your comment. We have added Figure 7 to report the 95% confidence intervals of the RMSE values across five independent training runs.

Comment 5: The study states that the model requires "only three basic battery parameters" (V_0, V_{EOD}, and C); however, the decoder uses C_{rated}, C_{EOL}, β, $r_{\min}$, and $r_{\max}$. This discrepancy needs to be clarified.

Response: Thank you for your comment. We revised the paragraph beginning with "We propose SeqBattNet, a discrete-state PINN for battery modeling ..." to improve clarity. In addition, β, $r_{\min}$, and $r_{\max}$ are treated as fixed parameters determined through grid search and are used to support the modeling of capacity degradation. This differs from previous approaches, where parameters should be remeasured or retuned through grid search to maintain accuracy as capacity degradation progresses.

Comment 6: There are also a few small grammatical errors. A thorough proofread would be beneficial for the manuscript.

Response: Thank you for your comment. We have reviewed the manuscript and corrected the grammatical errors.

Comment 7: There is inconsistent definition of some variables and dimensions, especially in the encoder formulation. Please make sure that every symbol is introduced in a clear and consistent manner.

Response: Thank you for your comment. We have revised the encoder formulation to clearly define all variables.

Comment 8: Equation: Not every equation is numbered, which makes it challenging to refer to particular steps.

Response: Thank you for your comment. We have added equation numbers to all equations.

To Reviewer 4

Comment 1: The comparison with BattNN, LSTM, GRU, Transformer, and encoder variants is useful. However, the authors should improve rigor by reporting repeated runs with mean $\pm$ standard deviation, random seeds, and preferably statistical significance tests or confidence intervals. This is important because deep learning results may vary across initialization and training conditions.

Response: Thank you for your comment. We have added Figure 7 and a corresponding paragraph beginning with "Fig. 7 shows the mean RMSE and the corresponding ..." to report the confidence intervals obtained from five independent training runs.

Comment 2: The manuscript states that TRI, RT-Batt, and NASA datasets are used, but the data availability statement should provide direct links, accession information, or DOIs. The authors should also consider sharing the preprocessing scripts, train/validation/test split files, hyperparameter settings, and source code to allow full reproducibility.

Response: Thank you for your comment. In the Dataset section, we have clarified data availability by adding direct links to the public datasets and providing detailed train/validation/test split information together with the corresponding dataset parameters in Table 3. However, we are unable to share the source code due to security reasons.

Comment 3: The proposed weighted loss and Hierarchical Reasoning–GRU encoder appear beneficial, but the paper would be stronger with clearer ablation analysis. The authors should explicitly quantify the contribution of the aging-adaptation parameters, the weighted loss, and the discrete-state formulation separately.

Response: Thank you for your comment. Regarding the proposed weighted loss, Table 4 provides a comparison between standard baseline loss functions and the proposed loss formulation, which quantifies its contribution to performance improvement.

For the Hierarchical Reasoning–GRU encoder used to generate aging-adaptation parameters, we clarified its contribution through comparison with BattNN, where fixed physical parameters are determined through grid search rather than learned adaptively from data. This comparison highlights the advantage of the proposed encoder in capturing cycle-specific degradation behavior.

Regarding the discrete-state formulation, replacing the current framework with a continuous ODE-based formulation would require redesigning the underlying physical equations and state-transition mechanism, resulting in a fundamentally different modeling framework rather than a direct ablation setting. Therefore, we consider continuous-state modeling as an important direction for future work and plan to investigate it in a separate study.

Comment 4: The authors correctly mention that temperature is not included. This limitation should be emphasized more strongly in the conclusion, because temperature strongly affects resistance, capacity fade, and voltage response. The manuscript should avoid implying broad real-world deployment before validation on datasets with varying temperature and operating conditions.

Response: Thank you for your comment. We have improved the Discussion and Limitations section and the Conclusion section to more explicitly emphasize the limitation of excluding temperature effects.

Comment 5: The paper is generally understandable, but several sentences need grammatical correction and polishing. Examples include phrases such as "It has a ability" and some long claims in the introduction and contribution section. The figures and tables are useful, but Table 4 is dense and should be reformatted for readability.

Response: Thank you for your comment. We have carefully reviewed the manuscript and corrected grammatical and language issues throughout the paper, including improving sentence clarity in the Introduction and Contribution sections. In addition, we revised several long sentences to improve readability.

Regarding Table 5 (Table 4 in the previous version), we considered multiple formatting alternatives; however, we found that the current format provides better readability. Since the table presents results from several models, datasets, and evaluation metrics simultaneously, we retained the current structure as the most effective balance between compactness and clarity.
