# Supplementary material for: SeqBattNet: A discrete-state physics-informed neural network with aging adaptation for battery modeling
Source: PLoS One. 2026 Nov 12;21(11):e0362362. doi: 10.1371/journal.pone.0362362 (PMC13759123; doi:10.1371/journal.pone.0362362)
Supplement: Supplementary file 2 [file pone.0362362.s003.pdf]

Response Letter

We sincerely thank Editor-in-Chief, Associate Editor, and the reviewers for their constructive, pertinent, and useful comments on our manuscript.

To Editor-in-Chief and Associate Editor

We are pleased to see insightful and constructive comments from the reviewers. In this resubmission, we have attempted to address the reviewers' concerns and revised the manuscript accordingly. Further details about all the changes we made can be found in our point-to-point response to the reviewers' comments below. In the revised manuscript, we marked the changes in yellow.

To Reviewer 1

Comment 1: First, the comparison with recent methods is still limited. The authors added more references, but no new state-of-the-art models were included in the experiments. They should either add suitable recent baselines or explain clearly why direct comparison is not possible.

Response: Thank you for your comment. We have addressed this concern by adding comparisons with two recent state-of-the-art physics-informed battery modeling methods, namely BattNN (2023) and PDB-Net (2025), in Table 6 and the corresponding discussion in the Comparison with State-of-the-Art Physics-Informed Battery Models subsection.

Comment 2: Second, the ablation study is incomplete. The weighted loss is tested, but the effects of aging adaptation and the discrete-state design are not isolated. A controlled comparison using fixed versus cycle-specific parameters would strengthen the paper. The authors should also report end-of-discharge timing error or related measures, since this is a main claimed advantage.

Response: Thank you for your comment. We have revise Table 4 and the corresponding discussion starting with "Table 4 compares the proposed loss function with the standard MSE loss ..." in the Aging Adaptation and Loss Function Ablation Study subsection. That compares MSE, BattNN, and our weighted loss function with and without aging adaptation.

A continuous-state comparison is not directly applicable because the discrete-state formulation is embedded in both the model architecture and the prediction process. Developing a matched continuous-state counterpart is therefore left for future work.

Moreover, this study focuses on battery modeling. End-of-discharge prediction additionally requires forecasting future current profiles, which will be investigated in future work, as noted in the paragraph beginning with "Additionally, the scope of this study is limited to battery modeling, ..." in the Discussion and Limitations subsection.

Comment 3: Third, the statistical reporting needs clarification. The paper should state the random seeds, explain how the five runs were combined, and report the mean plus standard deviation. Confidence intervals for SeqBattNet alone do not prove statistical significance over the baselines.

Response: Thank you for your comment. We have updated the paragraph beginning with "We use the default random-number initialization in NumPy and PyTorch ..." in the Experimental Setup subsection to clarify the random initialization, the procedure for combining the five experimentals runs, and the statistical reporting.

Comment 4: Reproducibility also remains a concern. The reason for not sharing code is vague. At a minimum, the authors should provide preprocessing steps, split files, baseline settings, random seeds, and full hyperparameter details.

Response: Thank you for your valuable comment. We have added several paragraphs addressing these concerns. Specifically, we now provide detailed descriptions of the preprocessing steps, data splits, baseline settings, random seeds, and hyperparameter configurations in the Experimental Setup subsection.

Comment 5: Finally, the generalization claims should be moderated. The results support cross-cell generalization within the tested datasets, not broad real-world use across chemistries, temperatures, and manufacturers.

Response: Thank you for your comment. We agree that our experiments demonstrate cross-cell generalization within the evaluated datasets rather than broad real-world generalization. Accordingly, we have revised the Abstract to moderate this claim that SeqBattNet demonstrates strong cross-cell generalization on the evaluated datasets.

To Reviewer 2

Comment 1: However, a significant issue remains: several references are weakly related or entirely irrelevant to the specific content they are cited for, which undermines the scholarly quality of the manuscript

Response: Thank you for your comment. We have carefully reviewed all citations throughout the manuscript. We revised AdamW citation and remove the citation of ReduceLROnPlateau.

To Reviewer 3

Comment 1: instead of listing limitations, the author should say like this why continuous-state PINNs fundamentally struggle with long-term battery degradation while discrete-state modeling is more suitable.

Response: Thank you for your comment. We have revised the Introduction to explain more clearly why continuous-state PINNs are less suitable for long-horizon battery degradation modeling and why a discrete-state formulation is more appropriate. Specifically, we added a discussion beginning with "Although continuous-state formulations effectively model short-term battery dynamics, ...".

Comment 2: this reference can be helpful about the gaps identifications: Second-Life Batteries as Enablers of Sustainable Electric Vehicle Infrastructure: Opportunities and Future Directions

Response: Thank you for your comment. We have reviewed the recommended reference and incorporated it into the Introduction section to strengthen the discussion of the research gaps. Specifically, we added a paragraph beginning with "Recent review studies have highlighted...".

Comment 3: this idea is repeated: generalizes across many cells

Response: Thank you for your comment. We have replaced the repeated statement regarding generalization across cells by alternatives to avoid repetition.

Comment 4: What aging parameters are being learned? Which variables most influence adaptation?

Response: Thank you for your comment. We have clarified that R_0, $\boldsymbol{\tau}$, SOC_0, SOH, and $\mathbf{v}_{RC,0}$ are the learned parameters used for aging adaptation in the proposed method. In addition, we have added Fig. 6 and the corresponding discussion to identify and validate the features that have the greatest influence on the model's adaptation using the validation dataset.

Comment 5: add atble to compre relevant studies with their approach, metrics, battery type

Response: Thank you for your comment. We have added Table 6 to compare our proposed method with other state-of-the-art methods on the same dataset.

Comment 6: add numerical results in the abstract and the conclusion, while depending on results add feasible future directions.

Response: Thank you for your comment. We have revised both the abstract and the conclusion by adding the key quantitative results of the proposed method.

Comment 7: figures 2 to 6 have low quality, increase the font size and axis.

Response: Thank you for your comment. We have revised Figures 2–6 by increasing the font sizes of the titles, axis labels, tick labels, and legends to improve readability.

Comment 8: defined variables like Crated ,CEOL, beta, r min, r max as the first time near the equations in the content.

Response: Thank you for your comment. These variables are already defined immediately after they are first introduced in the algorithm.

Comment 9: strong discussion is required in term of the results and approach used.

Response: Thank you for your comment. We have added more discussion in the Experimental section to provide a more comprehensive analysis of the proposed approach.
