# Supplementary material for: SeqBattNet: A discrete-state physics-informed neural network with aging adaptation for battery modeling
Source: PLoS One. 2026 Nov 12;21(11):e0362362. doi: 10.1371/journal.pone.0362362 (PMC13759123; doi:10.1371/journal.pone.0362362)
Supplement: Supplementary file 3 [file pone.0362362.s004.pdf]

Response Letter

We sincerely thank Editor-in-Chief, Associate Editor, and the reviewers for their constructive, pertinent, and useful comments on our manuscript.

To Editor-in-Chief and Associate Editor

We are pleased to see insightful and constructive comments from the reviewers. In this resubmission, we have attempted to address the reviewers' concerns and revised the manuscript accordingly. Further details about all the changes we made can be found in our point-to-point response to the reviewers' comments below. In the revised manuscript, we marked the changes in yellow.

To Reviewer 6

All the questions have been addressed well. I thank the authors for the prompt responses. I thus recommend it for publication as is.

Response: Thank you so much.

To Reviewer 7

1. Tables 4,5 should be in the next page and increase the font like the journal guidline or event your text.

Response: thank you for your comment. We have already used the largest possible font size while ensuring that the tables fit within the page margins. Additionally, Tables 4 and 5 are positioned close to their first mention in the text to maintain readability and coherence.

2. Some figures quality need to be improved and enlarge the size of figures while the font size of axis and legends needs to be increased.

Response: Thank you for your comment. We have increased the font sizes of the axis labels, tick labels, and other text elements in Fig. 8 to improve readability.

3. In equation 2, the matrix multiplication is dimensionally inconsistent.

Response: Thank you for your comment. We have corrected the matrix multiplication in Equation (2).

4. In equation 15, Separate symbols for V0 should be used.

Response: Thank you for your comment. We have revised Equations (11) and (15) to distinguish the voltage parameters.

5. Ceff in equation 16 should be defined before Algorithm 1 or included among the required decoder parameters.

Response: Thank you for your comment. We have revised Algorithm 1 to explicitly define the effective capacity (Ceff) before the iterative ECM updates.

6. Strong cross-cell generalization, is strong without test temperature or other chemistry types, rewrite in another words. Response: Thank you for your comment. We have revised the statement to clarify that the model demonstrates promising predictive performance on unseen battery cells within the evaluated datasets. The revised statement begins with "demonstrates promising predictive performance...".

7. Remove such claims: Robust and reliable voltage predictions, and also more suitable for real-world applications which is not adequately demonstrated because there is no embedded implementation, real-time latency analysis.

Response: Thank you for your comment. We have revised the manuscript to remove unsupported claims regarding robustness, reliability, and suitability for real-world applications. the revised statement begins with "These results demonstrate that SeqBattNet achieves accurate terminal.."

8. Avoiding vanishing gradients should be replaced with sentence such as mitigating the vanishing-gradient problem.

Response: Thank you for your comment. We have replaced "avoiding vanishing gradients" with "mitigating the vanishing-gradient problem".

To Reviewer 8

1. R0, tau, SOC0, SOH, and vRC,0 are learned from the voltage-prediction objective, without independent measurements or parameter identification. Fig. 6 shows that these quantities affect the predicted voltage, but this does not establish that their learned values are physically correct or uniquely identifiable. The description of these outputs as "physically meaningful parameters," particularly in Sections 5.3 and 6, should therefore be qualified.

Response: Thank you for your comment. We have revised Sections 5.3 and 6 to clarify that Figure 6 demonstrates parameter sensitivity rather than physical accuracy or identifiability. The revised statements begin with "These findings indicate that the encoder-generated..." in Section 5.3 and "The sensitivity analysis shows that..." in Section 6.

2. It is not fully clear how the five runs were used. Section 5.1 first states that their predictions were averaged to obtain the final prediction, but later states that the reported metrics are the averages of five independently calculated results. Please clarify which procedure was used in Tables 4–6. As the five runs have already been completed, the standard deviations for the main results could also be reported.

Response: Thank you for your comment. We have revised Section 5.1 to clarify the evaluation

procedure. The revised statement begins with "We use the default random-number initialization...". Since the standard deviations across the five runs are small, we have omitted them from Tables 4–6 to avoid overcrowding the tables. Instead, Figure 8 presents the 95% confidence intervals to illustrate the variability in the proposed model's performance.

3. The reported MAPE values appear to require correction. Equation (22) includes multiplication by 100, but Tables 4–6 seem to report the errors as fractions. For instance, an MAE of about 0.019 V for a voltage around 3–4 V would give a MAPE of roughly 0.5%, not 0.0055%. Please check the calculation and the "MAPE (%)" headings throughout.

Response: Thank you for your comment. We have revised the MAPE definition in Equation (21) to express the error as a fraction rather than a percentage. Accordingly, we have removed the percentage symbol from the MAPE headings in Tables 4–6.

4. The literature on physics-integrated battery learning is still somewhat limited. Relevant studies include but are not limited to "MINN: Learning the dynamics of differential-algebraic equations and application to battery modeling" and "BMINN: Learning chemical potentials and parameters from voltage data for multi-phase battery modeling."

Response: Thank you for your comment. We have expanded the literature review in Section 1 to include the MINN and BMINN studies. The revised paragraph begins with "Recent studies have further explored physics-integrated learning...".

5. There are still a few presentation issues. The RT-Batt heading repeats "Different Discharge Profiles for cells." The use of "Fig" and "Figure" is inconsistent, as is the capitalization in the dataset headings. RMSE, MAE, and MAPE are also written with unnecessary spaces between letters in several places.

Response: Thank you for your comment. We have corrected the duplicated RT-Batt heading, standardized the capitalization of dataset headings and figure references, and fixed the spacing of RMSE, MAE, and MAPE throughout the manuscript.

To Reviewer 9

1. The no-aging ablation uses parameters selected from initial cycles and then fixed, which also changes the fitting procedure. Please clarify which parameters are fixed and whether the remaining components are trained identically. This comparison supports the value of adaptation but does not fully isolate its contribution.

Response: Thank you for your comment. We have revised Section 5.3 to specify the fixed parameters and clarify that the remaining components retain the same training settings.

2. The explanation for omitting end-of-discharge timing error is not sufficient. Since the evaluation already supplies the future current profile, cutoff-crossing error can be assessed under that known profile without forecasting current. Please include this measure or narrow the related claims.

Response: Thank you for your comment. We agree that EOD timing error can be evaluated under a known future current profile. As this study focuses on battery modeling and terminal voltage prediction, we have revised Sections 1, 3.4, and 5.6 by adding the statements "The proposed discrete-state formulation enables sequential terminal...", "As this study focuses on battery voltage modeling...", and "This study focuses exclusively on battery modeling...", respectively, to clarify the study's scope and avoid unsupported EOD/RDT performance claims.
