# Supplementary material for: SeqBattNet: A discrete-state physics-informed neural network with aging adaptation for battery modeling
Source: PLoS One. 2026 Nov 12;21(11):e0362362. doi: 10.1371/journal.pone.0362362 (PMC13759123; doi:10.1371/journal.pone.0362362)
Supplement: Peer review history

# Peer review: Decision Letter 0

**DOI:** 10.1371/journal.pone.0362362.r001

**Authors:** Shih-Lin Lin

2 Jun 2026

Dear Dr. Trinh,

Thank you for submitting your manuscript to PLOS ONE. After careful consideration, we feel that it has merit but does not fully meet PLOS ONE’s publication criteria as it currently stands. Therefore, we invite you to submit a revised version of the manuscript that addresses the points raised during the review process.

Please submit your revised manuscript by Jul 17 2026 11:59PM. If you will need more time than this to complete your revisions, please reply to this message or contact the journal office at plosone@plos.org. When you're ready to submit your revision, log on to https://www.editorialmanager.com/pone/ and select the 'Submissions Needing Revision' folder to locate your manuscript file.

- A letter that responds to each point raised by the academic editor and reviewer(s). You should upload this letter as a separate file labeled 'Response to Reviewers'.
- A marked-up copy of your manuscript that highlights changes made to the original version. You should upload this as a separate file labeled 'Revised Manuscript with Track Changes'.
- An unmarked version of your revised paper without tracked changes. You should upload this as a separate file labeled 'Manuscript'.

If you would like to make changes to your financial disclosure, please include your updated statement in your cover letter. Guidelines for resubmitting your figure files are available below the reviewer comments at the end of this letter.

If applicable, we recommend that you deposit your laboratory protocols in protocols.io to enhance the reproducibility of your results. Protocols.io assigns your protocol its own identifier (DOI) so that it can be cited independently in the future. For instructions see: https://journals.plos.org/plosone/s/submission-guidelines#loc-laboratory-protocols. Additionally, PLOS ONE offers an option for publishing peer-reviewed Lab Protocol articles, which describe protocols hosted on protocols.io. Read more information on sharing protocols at https://plos.org/protocols?utm_medium=editorial-email&utm_source=authorletters&utm_campaign=protocols.

As the corresponding author, your ORCID iD is verified in the submission system and will appear in the published article. PLOS supports the use of ORCID, and we encourage all coauthors to register for an ORCID iD and use it as well. Please encourage your coauthors to verify their ORCID iD within the submission system before final acceptance, as unverified ORCID iDs will not appear in the published article. *Only*  the individual author can complete the verification step; PLOS staff *cannot*  verify ORCID iDs on behalf of authors.

We look forward to receiving your revised manuscript.

Kind regards,

Shih-Lin Lin, Ph.D

Academic Editor

PLOS One

Journal Requirements:

When submitting your revision, we need you to address these additional requirements.

1. Please ensure that your manuscript meets PLOS ONE's style requirements, including those for file naming. The PLOS ONE style templates can be found at

https://journals.plos.org/plosone/s/file?id=wjVg/PLOSOne_formatting_sample_main_body.pdf and

https://journals.plos.org/plosone/s/file?id=ba62/PLOSOne_formatting_sample_title_authors_affiliations.pdf.

2. Please note that PLOS One has specific guidelines on code sharing for submissions in which author-generated code underpins the findings in the manuscript. In these cases, we expect all author-generated code to be made available without restrictions upon publication of the work. Please review our guidelines at https://journals.plos.org/plosone/s/materials-and-software-sharing#loc-sharing-code and ensure that your code is shared in a way that follows best practice and facilitates reproducibility and reuse.

3. We note that the grant information you provided in the ‘Funding Information’ and ‘Financial Disclosure’ sections do not match.

When you resubmit, please ensure that you provide the correct grant numbers for the awards you received for your study in the ‘Funding Information’ section.

4. Please note that your Data Availability Statement is currently missing [the repository name and/or the DOI/accession number of each dataset OR a direct link to access each database]. If your manuscript is accepted for publication, you will be asked to provide these details on a very short timeline. We therefore suggest that you provide this information now, though we will not hold up the peer review process if you are unable.

5. If the reviewer comments include a recommendation to cite specific previously published works, please review and evaluate these publications to determine whether they are relevant and should be cited. There is no requirement to cite these works unless the editor has indicated otherwise.

Additional Editor Comments:

Reviewer-suggested references are not mandatory. Please include only references that are directly relevant to the manuscript and that help clarify the study’s scholarly context. Unrelated or marginally relevant citations should be avoided.

[Note: HTML markup is below. Please do not edit.]

Reviewers' comments:

Reviewer's Responses to Questions

**Comments to the Author**

1. Is the manuscript technically sound, and do the data support the conclusions?

Reviewer #1: Yes

Reviewer #2: Partly

Reviewer #3: Yes

Reviewer #4: Yes

**********

2. Has the statistical analysis been performed appropriately and rigorously?

Reviewer #1: Yes

Reviewer #2: No

Reviewer #3: Yes

Reviewer #4: Yes

**********

3. Have the authors made all data underlying the findings in their manuscript fully available?

The PLOS Data policy

Reviewer #1: No

Reviewer #2: Yes

Reviewer #3: Yes

Reviewer #4: Yes

**********

4. Is the manuscript presented in an intelligible fashion and written in standard English?

Reviewer #1: Yes

Reviewer #2: Yes

Reviewer #3: Yes

Reviewer #4: Yes

**********

Reviewer #1: The authors proposed a novel PINN for modelling battery with a focus on lithium ion battery. Given the interest and diverse applications of battery this presents a timely study. However there are few areas of interest that could be improved on. First a background of different battery modelling should be presented as well as if physics informed models have been used previously, Secondly, All images are not clear and should be improved. Additionally, section 4 on data set could do with a summary statistics and heat map. The section should also include a link to a publicly available data and code for the work.

In terms of the theoretical concepts, there are several equations that are readily available in literature and could be moved to supplementary information.

Reviewer #2: This paper introduces a hybrid framework for lithium-ion battery voltage prediction called SeqBattNet, which combines an aging-aware mechanism with a discrete-state physics-informed neural network (PINN). The method aims to improve generalization across battery cells while reducing parameter dependency by combining a physics-informed equivalent circuit model (ECM) decoder with a Hierarchical Reasoning–GRU encoder.

The paper has promising performance across several benchmark datasets and is technically sound. However, before the manuscript can be considered for publication, a number of significant issues pertaining to experimental validation, presentation clarity, and the rationale behind design decisions should be resolved.

1. The paper mainly contrasts SeqBattNet with a traditional sequence models (LSTM and GRU) and a small number of prior PINN-based methods. The review is weakened by the lack of comparisons with more recent state-of-the-art techniques because battery modeling and physics-informed machine learning are developing at a rapid pace. I recommend that you compare with more recent hybrid electrochemical-ML models or PINN-based models using relevant publications from the past two to three years.

2. I understand that you mentioned that temperature effect is not considered in your work, I will suggest that you expand discussion on how this limitation affects real-world deployment. If possible, include preliminary analysis or justification for excluding temperature.

3. You highlighted data leakage in previous works, however, your experimental design raises concern regarding training diversity. The model is specifically trained on a single cell per dataset (Lines 278–279, 287–288, 296–297), which restricts the training data's variability and might not be adequate to support the asserted generalization across many cells. Additionally, neither the number of cycles used for training nor testing nor whether operating conditions (such as discharge profiles or deterioration phases) overlap between splits are explicitly mentioned in the study. Because of this lack of information, it is challenging to evaluate the evaluation's impartiality and robustness. It is recommended that the authors publish more detailed information on the training and testing splits and make clear how the dataset is used.

4. The results reported in Tables 3 and 4 present only the mean performance metrics (RMSE, MAE, MAPE), despite the fact that each experiment is repeated multiple times (Lines 320–321). Reporting merely the mean could mask model performance variability since neural network training is stochastic. I urge that you provide the standard deviation (or confidence intervals) with the mean values in order to offer a more thorough and trustworthy assessment. This will assist in determining the stated improvements' stability and statistical significance.

5. T he study states that the model requires "only three basic battery parameters" (V₀, VEOD, and C), yet, the decoder uses Crated, CEOL, β, rmin, rmax. This discrepancy needs to be clarified.

6. There are also a few small grammatical errors. A thorough proofread would be beneficial for the manuscript.

7. There is inconsistent definition of some variables and dimensions, especially in the encoder formulation. Please make sure that every symbol is introduced in a clear and consistent manner.

8. Equation: Not every equation is numbered, which makes it challenging to refer to particular steps.

Reviewer #3: Accurate battery modeling is essential for reliable state estimation in modern applications, especially for predicting remaining discharge time and remaining discharge energy in battery management systems. However, existing approaches still face important limitations. Model-based methods often require a large number of parameters, data-driven methods depend heavily on labeled datasets, and many current physics-informed neural networks either lack aging adaptation, still involve too many parameters, or require continuous state regeneration. To address these challenges, this work proposes SeqBattNet, a discrete-state physics-informed neural network with built-in aging adaptation for battery modeling, designed to predict terminal voltage during the discharge process.

SeqBattNet is composed of two main parts. The first part is an encoder, implemented through the proposed Hierarchical Reasoning–GRU deep learning module, which generates cycle-specific aging adaptation parameters. The second part is a decoder, built on an equivalent circuit model combined with deep learning, which uses these generated parameters together with the input current to predict the battery voltage. A key advantage of the framework is that it requires only three basic battery parameters, yet when trained on data from a single cell, it still demonstrates robust performance across many other cells. This shows that the method has strong generalization ability while remaining computationally efficient and structurally compact.

The performance of SeqBattNet is evaluated on three benchmark datasets, namely TRI, RT-Batt, and NASA. The results show that it significantly outperforms classical sequence models and existing physics-informed neural network baselines, achieving consistently lower RMSE while maintaining good computational efficiency. These findings indicate that SeqBattNet provides a practical and effective solution for battery voltage prediction, especially in applications where aging adaptation, parameter efficiency, and cross-cell robustness are all important.

This work is closely aligned with ["An electric-thermal coupling modeling method for lithium-ion battery using the state of charge normalization calculation method", Journal of Energy Storage], where battery behavior is modeled with attention to internal condition dependence and practical prediction needs. The present study follows a similar direction by aiming for accurate and reliable state-related battery modeling, but strengthens it through discrete-state physics-informed learning and built-in aging adaptation.

It is also strongly connected to ["Remaining useful life prediction of lithium-ion batteries based on an incremental internal resistance aging model and a gated recurrent unit neural network", Energy], which highlights the importance of capturing aging-related battery behavior through GRU-based learning mechanisms. The current work shares this aging-sensitive perspective and further integrates it into a physics-informed battery voltage prediction framework.

A similar methodological relevance can be seen in ["An asynchronous electro-thermal coupling modeling method of lithium-ion batteries under dynamic operating conditions", Energy], where accurate battery behavior prediction under realistic operating conditions is emphasized. In the same broader context, SeqBattNet aims to improve predictive accuracy under discharge conditions while preserving computational practicality.

Finally, the overall modeling philosophy is consistent with ["A review of equivalent-circuit model, degradation characteristics and economics of Li-ion battery energy storage system for grid applications", Journal of Energy Storage], which underlines the value of equivalent-circuit-based battery representation for practical monitoring and control. The present study builds on that broader idea by combining ECM structure with deep learning and physics-informed design to achieve accurate, aging-adaptive, and efficient battery modeling.

Reviewer #4: The manuscript presents SeqBattNet, a discrete-state physics-informed neural network with aging adaptation for terminal voltage prediction in lithium-ion batteries. The topic is relevant, and the proposed combination of an aging-adaptive encoder with an ECM-based decoder is technically interesting. The use of three public datasets with different discharge-profile characteristics is a strength, and the reported results show clear improvements over several baselines.

However, I recommend minor revision before publication.

Technical soundness and conclusions:

The overall methodology is reasonable, and the conclusions are mostly supported by the reported RMSE, MAE, and MAPE results. However, some claims should be softened, especially those related to generalization and real-world applicability, since the study excludes temperature effects and uses laboratory datasets only.

Statistical and experimental rigor:

The comparison with BattNN, LSTM, GRU, Transformer, and encoder variants is useful. However, the authors should improve rigor by reporting repeated runs with mean ± standard deviation, random seeds, and preferably statistical significance tests or confidence intervals. This is important because deep learning results may vary across initialization and training conditions.

Data and reproducibility:

The manuscript states that TRI, RT-Batt, and NASA datasets are used, but the data availability statement should provide direct links, accession information, or DOIs. The authors should also consider sharing the preprocessing scripts, train/validation/test split files, hyperparameter settings, and source code to allow full reproducibility.

Ablation and model justification:

The proposed weighted loss and Hierarchical Reasoning–GRU encoder appear beneficial, but the paper would be stronger with clearer ablation analysis. The authors should explicitly quantify the contribution of the aging-adaptation parameters, the weighted loss, and the discrete-state formulation separately.

Limitations:

The authors correctly mention that temperature is not included. This limitation should be emphasized more strongly in the conclusion, because temperature strongly affects resistance, capacity fade, and voltage response. The manuscript should avoid implying broad real-world deployment before validation on datasets with varying temperature and operating conditions.

Writing and presentation:

The paper is generally understandable, but several sentences need grammatical correction and polishing. Examples include phrases such as “It has a ability” and some long claims in the introduction and contribution section. The figures and tables are useful, but Table 4 is dense and should be reformatted for readability.

**********

what does this mean?). If published, this will include your full peer review and any attached files.

If you choose “no”, your identity will remain anonymous but your review may still be made public.

**Do you want your identity to be public for this peer review?**  For information about this choice, including consent withdrawal, please see our Privacy Policy

Reviewer #1: No

Reviewer #2: No

Reviewer #3: No

Reviewer #4: No

**********

[NOTE: If reviewer comments were submitted as an attachment file, they will be attached to this email and accessible via the submission site. Please log into your account, locate the manuscript record, and check for the action link "View Attachments". If this link does not appear, there are no attachment files.]

To ensure your figures meet our technical requirements, please review our figure guidelines: https://journals.plos.org/plosone/s/figures

You may also use PLOS’s free figure tool, NAAS, to help you prepare publication quality figures: https://journals.plos.org/plosone/s/figures#loc-tools-for-figure-preparation.

NAAS will assess whether your figures meet our technical requirements by comparing each figure against our figure specifications.

# Author comment: Author response to Decision Letter 1

**DOI:** 10.1371/journal.pone.0362362.r002

11 Jun 2026

Response Letter

We sincerely thank Editor-in-Chief, Associate Editor, and the reviewers for their constructive,

pertinent, and useful comments on our manuscript.

To Editor-in-Chief and Associate Editor

We are pleased to see insightful and constructive comments from the reviewers. In this resubmission, we have attempted to address the reviewers’ concerns and revised the manuscript

accordingly. Further details about all the changes we made can be found in our point-to-point

response to the reviewers’ comments below. In the revised manuscript, we marked the changes

in yellow.

To Reviewer 1

A background of different battery modelling should be presented as well as if physics informed

models have been used previously.

Response: Thank you for your comment. We have expanded the Introduction section to provide

additional background on battery modelling approaches. We have added a discussion of existing

PINN-based battery modelling studies and clarified the motivation for using an ECM-based

physics-informed formulation for RDT prediction.

Comment 2: All images are not clear and should be improved

Response: Thank you for your comment. Figure 1, which presents the overall architecture of the

proposed SeqBattNet, has been improved to enhance clarity and readability.

Comment 3: section 4 on data set could do with a summary statistics and heat map. The section

should also include a link to a publicly available data and code for the work.

Response: Thank you for your comment. We have added summary statistics and correlation

heat maps for the three datasets. We have also included links to the public datasets. However,

we cannot share the code for security reasons.

To Reviewer 2

Comment 1: The paper mainly contrasts SeqBattNet with a traditional sequence models (LSTM

and GRU) and a small number of prior PINN-based methods. The review is weakened by the

lack of comparisons with more recent state-of-the-art techniques because battery modeling and

physics-informed machine learning are developing at a rapid pace. I recommend that you compare with more recent hybrid electrochemical-ML models or PINN-based models using relevant

1

publications from the past two to three years.

Response: Thank you for your comment. We have added more background information and included additional discussion of previous studies on PINNs in battery modeling in the Introduction

section.

Comment 2: I understand that you mentioned that temperature effect is not considered in

your work, I will suggest that you expand discussion on how this limitation affects real-world

deployment. If possible, include preliminary analysis or justification for excluding temperature.

Response: Thank you for your comment. We have expanded the discussion in the Discussion

and Limitations section to clarify the impact of excluding temperature effects on real-world

deployment.

Comment 3: You highlighted data leakage in previous works, however, your experimental design

raises concern regarding training diversity. The model is specifically trained on a single cell per

dataset (Lines 278–279, 287–288, 296–297), which restricts the training data’s variability and

might not be adequate to support the asserted generalization across many cells. Additionally,

neither the number of cycles used for training nor testing nor whether operating conditions (such

as discharge profiles or deterioration phases) overlap between splits are explicitly mentioned in

the study. Because of this lack of information, it is challenging to evaluate the evaluation’s impartiality and robustness. It is recommended that the authors publish more detailed information

on the training and testing splits and make clear how the dataset is used.

Response: Thank you for your comment. We added Table 3, which summarizes the dataset characteristics and provides detailed train/validation/test split configurations, including the selected

cells and the number of cycles used in each split. In this study, we intentionally trained the

model using data from a single battery cell and evaluated it on unseen cells for validation and

testing to assess its ability to generalize across different degradation trajectories using only limited training data. This setup was chosen to reflect practical scenarios, where obtaining labeled

battery degradation data is costly and time-consuming. This rationale has been clarified in the

paragraph above Table 3.

Comment 4: The results reported in Tables 3 and 4 present only the mean performance metrics

(RMSE, MAE, MAPE), despite the fact that each experiment is repeated multiple times (Lines

320–321). Reporting merely the mean could mask model performance variability since neural

network training is stochastic. I urge that you provide the standard deviation (or confidence

intervals) with the mean values in order to offer a more thorough and trustworthy assessment.

This will assist in determining the stated improvements’ stability and statistical significance.

Response: Thank you for your comment. We have added Figure 7 to report the 95% confidence

intervals of the RMSE values across five independent training runs.

Comment 5: The study states that the model requires "only three basic battery parameters"

(V0, VEOD, and C); however, the decoder uses Crated, CEOL, β, rmin, and rmax. This discrepancy

needs to be clarified.

2

Response: Thank you for your comment. We revised the paragraph beginning with “We propose

SeqBattNet, a discrete-state PINN for battery modeling ...” to improve clarity. In addition, β,

rmin, and rmax are treated as fixed parameters determined through grid search and are used

to support the modeling of capacity degradation. This differs from previous approaches, where

parameters should be remeasured or retuned through grid search to maintain accuracy as capacity

degradation progresses.

Comment 6: There are also a few small grammatical errors. A thorough proofread would be

beneficial for the manuscript.

Response: Thank you for your comment. We have reviewed the manuscript and corrected the

grammatical errors.

Comment 7: There is inconsistent definition of some variables and dimensions, especially in the

encoder formulation. Please make sure that every symbol is introduced in a clear and consistent

manner.

Response: Thank you for your comment. We have revised the encoder formulation to clearly

define all variables.

Comment 8: Equation: Not every equation is numbered, which makes it challenging to refer to

particular steps.

Response: Thank you for your comment. We have added equation numbers to all equations.

To Reviewer 4

Comment 1: The comparison with BattNN, LSTM, GRU, Transformer, and encoder variants

is useful. However, the authors should improve rigor by reporting repeated runs with mean

± standard deviation, random seeds, and preferably statistical significance tests or confidence

intervals. This is important because deep learning results may vary across initialization and

training conditions.

Response: Thank you for your comment. We have added Figure 7 and a corresponding paragraph

beginning with "Fig. 7 shows the mean RMSE and the corresponding ..." to report the confidence

intervals obtained from five independent training runs.

Comment 2: The manuscript states that TRI, RT-Batt, and NASA datasets are used, but the

data availability statement should provide direct links, accession information, or DOIs. The

authors should also consider sharing the preprocessing scripts, train/validation/test split files,

hyperparameter settings, and source code to allow full reproducibility.

Response: Thank you for your comment. In the Dataset section, we have clarified data availability by adding direct links to the public datasets and providing detailed train/validation/test

split information together with the corresponding dataset parameters in Table 3. However, we

are unable to share the source code due to security reasons.

3

Comment 3: The proposed weighted loss and Hierarchical Reasoning–GRU encoder appear beneficial, but the paper would be stronger with clearer ablation analysis. The authors should

explicitly quantify the contribution of the aging-adaptation parameters, the weighted loss, and

the discrete-state formulation separately.

Response: Thank you for your comment. Regarding the proposed weighted loss, Table 4 provides

a comparison between standard baseline loss functions and the proposed loss formulation, which

quantifies its contribution to performance improvement.

For the Hierarchical Reasoning–GRU encoder used to generate aging-adaptation parameters,

we clarified its contribution through comparison with BattNN, where fixed physical parameters

are determined through grid search rather than learned adaptively from data. This comparison

highlights the advantage of the proposed encoder in capturing cycle-specific degradation behavior.

Regarding the discrete-state formulation, replacing the current framework with a continuous

ODE-based formulation would require redesigning the underlying physical equations and statetransition mechanism, resulting in a fundamentally different modeling framework rather than

a direct ablation setting. Therefore, we consider continuous-state modeling as an important

direction for future work and plan to investigate it in a separate study.

Comment 4: The authors correctly mention that temperature is not included. This limitation

should be emphasized more strongly in the conclusion, because temperature strongly affects

resistance, capacity fade, and voltage response. The manuscript should avoid implying broad

real-world deployment before validation on datasets with varying temperature and operating

conditions.

Response: Thank you for your comment. We have improved the Discussion and Limitations

section and the Conclusion section to more explicitly emphasize the limitation of excluding

temperature effects.

Comment 5: The paper is generally understandable, but several sentences need grammatical

correction and polishing. Examples include phrases such as “It has a ability” and some long

claims in the introduction and contribution section. The figures and tables are useful, but Table

4 is dense and should be reformatted for readability.

Response: Thank you for your comment. We have carefully reviewed the manuscript and corrected grammatical and language issues throughout the paper, including improving sentence

clarity in the Introduction and Contribution sections. In addition, we revised several long sentences to improve readability.

Regarding Table 5 (Table 4 in the previous version), we considered multiple formatting alternatives; however, we found that the current format provides better readability. Since the table

presents results from several models, datasets, and evaluation metrics simultaneously, we retained

the current structure as the most effective balance between compactness and clarity.

4

Attachment Submitted filename: Response_to_Reviewers.pdf

# Peer review: Decision Letter 1

**DOI:** 10.1371/journal.pone.0362362.r003

**Authors:** Shih-Lin Lin

14 Jul 2026

Dear Dr. Trinh,

Thank you for submitting your manuscript to PLOS ONE. After careful consideration, we feel that it has merit but does not fully meet PLOS ONE’s publication criteria as it currently stands. Therefore, we invite you to submit a revised version of the manuscript that addresses the points raised during the review process.

Please submit your revised manuscript by Aug 28 2026 11:59PM. If you will need more time than this to complete your revisions, please reply to this message or contact the journal office at plosone@plos.org. When you're ready to submit your revision, log on to https://www.editorialmanager.com/pone/ and select the 'Submissions Needing Revision' folder to locate your manuscript file.

- A letter that responds to each point raised by the academic editor and reviewer(s). You should upload this letter as a separate file labeled 'Response to Reviewers'.
- A marked-up copy of your manuscript that highlights changes made to the original version. You should upload this as a separate file labeled 'Revised Manuscript with Track Changes'.
- An unmarked version of your revised paper without tracked changes. You should upload this as a separate file labeled 'Manuscript'.

If you would like to make changes to your financial disclosure, please include your updated statement in your cover letter. Guidelines for resubmitting your figure files are available below the reviewer comments at the end of this letter.

If applicable, we recommend that you deposit your laboratory protocols in protocols.io to enhance the reproducibility of your results. Protocols.io assigns your protocol its own identifier (DOI) so that it can be cited independently in the future. For instructions see: https://journals.plos.org/plosone/s/submission-guidelines#loc-laboratory-protocols. Additionally, PLOS ONE offers an option for publishing peer-reviewed Lab Protocol articles, which describe protocols hosted on protocols.io. Read more information on sharing protocols at https://plos.org/protocols?utm_medium=editorial-email&utm_source=authorletters&utm_campaign=protocols.

As the corresponding author, your ORCID iD is verified in the submission system and will appear in the published article. PLOS supports the use of ORCID, and we encourage all coauthors to register for an ORCID iD and use it as well. Please encourage your coauthors to verify their ORCID iD within the submission system before final acceptance, as unverified ORCID iDs will not appear in the published article. *Only*  the individual author can complete the verification step; PLOS staff *cannot*  verify ORCID iDs on behalf of authors.

We look forward to receiving your revised manuscript.

Kind regards,

Academic Editor

PLOS One

Journal Requirements:

If the reviewer comments include a recommendation to cite specific previously published works, please review and evaluate these publications to determine whether they are relevant and should be cited. There is no requirement to cite these works unless the editor has indicated otherwise.

[Note: HTML markup is below. Please do not edit.]

Reviewers' comments:

Reviewer's Responses to Questions

**Comments to the Author**

Reviewer #5: (No Response)

Reviewer #6: (No Response)

Reviewer #7: (No Response)

**********

2. Is the manuscript technically sound, and do the data support the conclusions?

Reviewer #5: No

Reviewer #6: Partly

Reviewer #7: Partly

**********

3. Has the statistical analysis been performed appropriately and rigorously?

Reviewer #5: No

Reviewer #6: No

Reviewer #7: Yes

**********

4. Have the authors made all data underlying the findings in their manuscript fully available?

The PLOS Data policy

Reviewer #5: No

Reviewer #6: No

Reviewer #7: Yes

**********

5. Is the manuscript presented in an intelligible fashion and written in standard English?

Reviewer #5: Yes

Reviewer #6: Yes

Reviewer #7: Yes

**********

Reviewer #5: The revised manuscript is clearer and better organized. The authors have expanded the background, added dataset details and split information, numbered the equations, reported confidence intervals, and discussed the limitation of excluding temperature. Using different cells for training, validation, and testing is also a strong point because it reduces data leakage.

However, several concerns remain.

First, the comparison with recent methods is still limited. The authors added more references, but no new state-of-the-art models were included in the experiments. They should either add suitable recent baselines or explain clearly why direct comparison is not possible.

Second, the ablation study is incomplete. The weighted loss is tested, but the effects of aging adaptation and the discrete-state design are not isolated. A controlled comparison using fixed versus cycle-specific parameters would strengthen the paper. The authors should also report end-of-discharge timing error or related measures, since this is a main claimed advantage.

Third, the statistical reporting needs clarification. The paper should state the random seeds, explain how the five runs were combined, and report the mean plus standard deviation. Confidence intervals for SeqBattNet alone do not prove statistical significance over the baselines.

Reproducibility also remains a concern. The reason for not sharing code is vague. At a minimum, the authors should provide preprocessing steps, split files, baseline settings, random seeds, and full hyperparameter details.

Finally, the generalization claims should be moderated. The results support cross-cell generalization within the tested datasets, not broad real-world use across chemistries, temperatures, and manufacturers.

Minor issues include unclear MAPE units, reuse of the symbol beta, inconsistent Figure 3 labels, and the need for further language editing.

Overall, the work is promising, but a major revision is still needed.

Reviewer #6: The authors have made a substantial effort in this revision to address the major concerns raised by the reviewers. Notable improvements include enhanced experimental rigor (addition of confidence intervals), improved data availability statements (direct dataset links), clearer ablation discussion, and a more explicit acknowledgment of limitations. The technical content and presentation have both been strengthened. However, a significant issue remains: several references are weakly related or entirely irrelevant to the specific content they are cited for, which undermines the scholarly quality of the manuscript

Reviewer #7: here is my comments:

1- instead of listing limitations, the author should say like this why continuous-state PINNs fundamentally struggle with long-term battery degradation while discrete-state modeling is more suitable.

2- this reference can be helpful about the gaps identifications: Second-Life Batteries as Enablers of Sustainable Electric Vehicle Infrastructure: Opportunities and Future Directions

3- this idea is repeated: generalizes across many cells

4- What aging parameters are being learned?Which variables most influence adaptation?

5- add atble to compre relevant studies with their approach, metrics, battery type

6- add numerical results in the abstract and the conclusion, while depending on results add feasible future directions.

7- figures 2 to 6 have low quality, increase the font size and axis.

8- defined variables like Crated ,CEOL, beta, r min, r max as the first time near the equations in the content.

9- strong discussion is required in term of the results and approach used.

apply tthe comments in the paper not only answering me

**********

what does this mean?). If published, this will include your full peer review and any attached files.

If you choose “no”, your identity will remain anonymous but your review may still be made public.

**Do you want your identity to be public for this peer review?**  For information about this choice, including consent withdrawal, please see our Privacy Policy

Reviewer #5: No

Reviewer #6: No

Reviewer #7: No

**********

[NOTE: If reviewer comments were submitted as an attachment file, they will be attached to this email and accessible via the submission site. Please log into your account, locate the manuscript record, and check for the action link "View Attachments". If this link does not appear, there are no attachment files.]

To ensure your figures meet our technical requirements, please review our figure guidelines: https://journals.plos.org/plosone/s/figures

You may also use PLOS’s free figure tool, NAAS, to help you prepare publication quality figures: https://journals.plos.org/plosone/s/figures#loc-tools-for-figure-preparation.

NAAS will assess whether your figures meet our technical requirements by comparing each figure against our figure specifications.

# Author comment: Author response to Decision Letter 2

**DOI:** 10.1371/journal.pone.0362362.r004

21 Jul 2026

Response Letter

We sincerely thank Editor-in-Chief, Associate Editor, and the reviewers for their constructive,

pertinent, and useful comments on our manuscript.

To Editor-in-Chief and Associate Editor

We are pleased to see insightful and constructive comments from the reviewers. In this resubmission, we have attempted to address the reviewers’ concerns and revised the manuscript

accordingly. Further details about all the changes we made can be found in our point-to-point

response to the reviewers’ comments below. In the revised manuscript, we marked the changes

in yellow.

To Reviewer 1

Comment 1: First, the comparison with recent methods is still limited. The authors added more

references, but no new state-of-the-art models were included in the experiments. They should

either add suitable recent baselines or explain clearly why direct comparison is not possible.

Response: Thank you for your comment. We have addressed this concern by adding comparisons

with two recent state-of-the-art physics-informed battery modeling methods, namely BattNN

(2023) and PDB-Net (2025), in Table 6 and the corresponding discussion in the Comparison

with State-of-the-Art Physics-Informed Battery Models subsection.

Comment 2: Second, the ablation study is incomplete. The weighted loss is tested, but the effects

of aging adaptation and the discrete-state design are not isolated. A controlled comparison using

fixed versus cycle-specific parameters would strengthen the paper. The authors should also report

end-of-discharge timing error or related measures, since this is a main claimed advantage.

Response: Thank you for your comment. We have revise Table 4 and the corresponding discussion

starting with "Table 4 compares the proposed loss function with the standard MSE loss ..." in the

Aging Adaptation and Loss Function Ablation Study subsection. That compares MSE, BattNN,

and our weighted loss function with and without aging adaptation.

A continuous-state comparison is not directly applicable because the discrete-state formulation

is embedded in both the model architecture and the prediction process. Developing a matched

continuous-state counterpart is therefore left for future work.

Moreover, this study focuses on battery modeling. End-of-discharge prediction additionally requires forecasting future current profiles, which will be investigated in future work, as noted

in the paragraph beginning with "Additionally, the scope of this study is limited to battery

modeling, ..." in the Discussion and Limitations subsection.

Comment 3: Third, the statistical reporting needs clarification. The paper should state the

random seeds, explain how the five runs were combined, and report the mean plus standard

deviation. Confidence intervals for SeqBattNet alone do not prove statistical significance over

the baselines.

Response: Thank you for your comment. We have updated the paragraph beginning with "We

use the default random-number initialization in NumPy and PyTorch ..." in the Experimental Setup subsection to clarify the random initialization, the procedure for combining the five

experimentals runs, and the statistical reporting.

Comment 4: Reproducibility also remains a concern. The reason for not sharing code is vague.

At a minimum, the authors should provide preprocessing steps, split files, baseline settings,

random seeds, and full hyperparameter details.

Response: Thank you for your valuable comment. We have added several paragraphs addressing

these concerns. Specifically, we now provide detailed descriptions of the preprocessing steps, data

splits, baseline settings, random seeds, and hyperparameter configurations in the Experimental

Setup subsection.

Comment 5: Finally, the generalization claims should be moderated. The results support crosscell generalization within the tested datasets, not broad real-world use across chemistries, temperatures, and manufacturers.

Response: Thank you for your comment. We agree that our experiments demonstrate crosscell generalization within the evaluated datasets rather than broad real-world generalization.

Accordingly, we have revised the Abstract to moderate this claim that SeqBattNet demonstrates

strong cross-cell generalization on the evaluated datasets.

To Reviewer 2

Comment 1: However, a significant issue remains: several references are weakly related or entirely

irrelevant to the specific content they are cited for, which undermines the scholarly quality of

the manuscript

Response: Thank you for your comment. We have carefully reviewed all citations throughout

the manuscript. We revised AdamW citation and remove the citation of ReduceLROnPlateau.

To Reviewer 3

Comment 1: instead of listing limitations, the author should say like this why continuous-state

PINNs fundamentally struggle with long-term battery degradation while discrete-state modeling

is more suitable.

Response: Thank you for your comment. We have revised the Introduction to explain more

clearly why continuous-state PINNs are less suitable for long-horizon battery degradation modeling and why a discrete-state formulation is more appropriate. Specifically, we added a discussion

beginning with "Although continuous-state formulations effectively model short-term battery

dynamics, ...".

Comment 2: this reference can be helpful about the gaps identifications: Second-Life Batteries

as Enablers of Sustainable Electric Vehicle Infrastructure: Opportunities and Future Directions

Response: Thank you for your comment. We have reviewed the recommended reference and

incorporated it into the Introduction section to strengthen the discussion of the research gaps.

Specifically, we added a paragraph beginning with "Recent review studies have highlighted...".

Comment 3: this idea is repeated: generalizes across many cells

Response: Thank you for your comment. We have replaced the repeated statement regarding

generalization across cells by alternatives to avoid repetition.

Comment 4: What aging parameters are being learned? Which variables most influence adaptation?

Response: Thank you for your comment. We have clarified that R0, τ , SOC0, SOH, and vRC,0

are the learned parameters used for aging adaptation in the proposed method. In addition, we

have added Fig. 6 and the corresponding discussion to identify and validate the features that

have the greatest influence on the model’s adaptation using the validation dataset.

Comment 5: add atble to compre relevant studies with their approach, metrics, battery type

Response: Thank you for your comment. We have added Table 6 to compare our proposed

method with other state-of-the-art methods on the same dataset.

Comment 6: add numerical results in the abstract and the conclusion, while depending on results

add feasible future directions.

Response: Thank you for your comment. We have revised both the abstract and the conclusion

by adding the key quantitative results of the proposed method.

Comment 7: figures 2 to 6 have low quality, increase the font size and axis.

Response: Thank you for your comment. We have revised Figures 2–6 by increasing the font

sizes of the titles, axis labels, tick labels, and legends to improve readability.

Comment 8: defined variables like Crated ,CEOL, beta, r min, r max as the first time near the

equations in the content.

Response: Thank you for your comment. These variables are already defined immediately after

they are first introduced in the algorithm.

Comment 9: strong discussion is required in term of the results and approach used.

Response: Thank you for your comment. We have added more discussion in the Experimental

section to provide a more comprehensive analysis of the proposed approach.

Attachment Submitted filename: Response_to_Reviewers_auresp_2.pdf

# Peer review: Decision Letter 2

**DOI:** 10.1371/journal.pone.0362362.r005

**Authors:** Shih-Lin Lin

8 Oct 2026

Dear Dr. Trinh,

Thank you for submitting your manuscript to PLOS One. After careful consideration, we feel that it has merit but does not fully meet PLOS One’s publication criteria as it currently stands. Therefore, we invite you to submit a revised version of the manuscript that addresses the points raised during the review process.

Please submit your revised manuscript by Nov 22 2026 11:59PM. If you will need more time than this to complete your revisions, please reply to this message or contact the journal office at plosone@plos.org. When you're ready to submit your revision, log on to https://www.editorialmanager.com/pone/ and select the 'Submissions Needing Revision' folder to locate your manuscript file.

- A letter that responds to each point raised by the academic editor and reviewer(s). You should upload this letter as a separate file labeled 'Response to Reviewers'.
- A marked-up copy of your manuscript that highlights changes made to the original version. You should upload this as a separate file labeled 'Revised Manuscript with Track Changes'.
- An unmarked version of your revised paper without tracked changes. You should upload this as a separate file labeled 'Manuscript'.

If you would like to make changes to your financial disclosure, please include your updated statement in your cover letter. Guidelines for resubmitting your figure files are available below the reviewer comments at the end of this letter.

If applicable, we recommend that you deposit your laboratory protocols in protocols.io to enhance the reproducibility of your results. Protocols.io assigns your protocol its own identifier (DOI) so that it can be cited independently in the future. For instructions see: https://journals.plos.org/plosone/s/submission-guidelines#loc-laboratory-protocols. Additionally, PLOS One offers an option for publishing peer-reviewed Lab Protocol articles, which describe protocols hosted on protocols.io. Read more information on sharing protocols at https://plos.org/protocols?utm_medium=editorial-email&utm_source=authorletters&utm_campaign=protocols.

As the corresponding author, your ORCID iD is verified in the submission system and will appear in the published article. PLOS supports the use of ORCID, and we encourage all coauthors to register for an ORCID iD and use it as well. Please encourage your coauthors to verify their ORCID iD within the submission system before final acceptance, as unverified ORCID iDs will not appear in the published article. *Only*  the individual author can complete the verification step; PLOS staff *cannot*  verify ORCID iDs on behalf of authors.

We look forward to receiving your revised manuscript.

Kind regards,

Shih-Lin Lin, Ph.D

Academic Editor

PLOS One

Journal Requirements:

If the reviewer comments include a recommendation to cite specific previously published works, please review and evaluate these publications to determine whether they are relevant and should be cited. There is no requirement to cite these works unless the editor has indicated otherwise.

Please review your reference list to ensure that it is complete and correct. If you have cited papers that have been retracted, please include the rationale for doing so in the manuscript text, or remove these references and replace them with relevant current references. Any changes to the reference list should be mentioned in the rebuttal letter that accompanies your revised manuscript. If you need to cite a retracted article, indicate the article’s retracted status in the References list and also include a citation and full reference for the retraction notice.

[Note: HTML markup is below. Please do not edit.]

Reviewers' comments:

Reviewer's Responses to Questions

**Comments to the Author**

Reviewer #6: All comments have been addressed

Reviewer #7: All comments have been addressed

Reviewer #8: (No Response)

Reviewer #9: All comments have been addressed

**********

2. Is the manuscript technically sound, and do the data support the conclusions?

Reviewer #6: Yes

Reviewer #7: Yes

Reviewer #8: (No Response)

Reviewer #9: Yes

**********

3. Has the statistical analysis been performed appropriately and rigorously?

Reviewer #6: Yes

Reviewer #7: Yes

Reviewer #8: (No Response)

Reviewer #9: Yes

**********

4. Have the authors made all data underlying the findings in their manuscript fully available?

The PLOS Data policy

Reviewer #6: Yes

Reviewer #7: Yes

Reviewer #8: (No Response)

Reviewer #9: Yes

**********

5. Is the manuscript presented in an intelligible fashion and written in standard English?

Reviewer #6: Yes

Reviewer #7: Yes

Reviewer #8: (No Response)

Reviewer #9: Yes

**********

Reviewer #6: All the questions have been addressed well. I thank the authors for the prompt responses. I thus recommend it for publication as is.

Reviewer #7: dear author, my recommendation help you to enhance the paper quality and impact:

1- Tables 4,5 should be in the next page and increase the font like the journal guidline or event your text.

2- some figures qulaity need to be improved and enlarge the size of figures while the font size of axis and legends needs to be increased.

3- in equation 2 the matrix multiplication is dimensionally inconsistent.

4- in equaation 15 Separate symbols for V0 should be used.

5- Ceff in equation 16 should be defined before Algorithm 1 or included among the required decoder parameters.

6- Strong cross-cell generalization, is strong without test temoreture or other chemistry types, rewrite in another words.

7- remove such claims:Robust and reliable voltage predictions, and also more suitable for real-world applications which is not adequately demonstrated because there is no embedded implementation, real-time latency analysis.

8- Avoiding vanishing gradients should be replaced with sentence such as mitigating the vanishing-gradient problem.

please consider my comments as recommendations to enhance the paper quality.

Reviewer #8: I did not review early versions of this manuscript and this is the first time for me to read it. In my view, the paper is close to being publishable. I have only a few comments.

1. R_0, tau, SOC0, SOH, and v_RC,0 are learned from the voltage-prediction objective, without independent measurements or parameter identification. Fig. 6 shows that these quantities affect the predicted voltage, but this does not establish that their learned values are physically correct or uniquely identifiable. The description of these outputs as “physically meaningful parameters,” particularly in Sections 5.3 and 6, should therefore be qualified.

2. It is not fully clear how the five runs were used. Section 5.1 first states that their predictions were averaged to obtain the final prediction, but later states that the reported metrics are the averages of five independently calculated results. Please clarify which procedure was used in Tables 4–6. As the five runs have already been completed, the standard deviations for the main results could also be reported.

3. The reported MAPE values appear to require correction. Equation (22) includes multiplication by 100, but Tables 4–6 seem to report the errors as fractions. For instance, an MAE of about 0.019 V for a voltage around 3–4 V would give a MAPE of roughly 0.5%, not 0.0055%. Please check the calculation and the “MAPE (%)” headings throughout.

4. The literature on physics-integrated battery learning is still somewhat limited. Relevant studies include but are not limited to “MINN: Learning the dynamics of differential-algebraic equations and application to battery modeling” and “BMINN: Learning chemical potentials and parameters from voltage data for multi-phase battery modeling.”

5. There are still a few presentation issues. The RT-Batt heading repeats “Different Discharge Profiles for cells.” The use of “Fig” and “Figure” is inconsistent, as is the capitalization in the dataset headings. RMSE, MAE, and MAPE are also written with unnecessary spaces between letters in several places.

Reviewer #9: Thanks for the revision. The papers look good with only 2 minor concerns.

The revision adds useful baseline comparisons and an aging-adaptation ablation, and the generalization claims are more measured. However, 2 minor concerns might need to be resolved.

1. The no-aging ablation uses parameters selected from initial cycles and then fixed, which also changes the fitting procedure. Please clarify which parameters are fixed and whether the remaining components are trained identically. This comparison supports the value of adaptation but does not fully isolate its contribution.

2. The explanation for omitting end-of-discharge timing error is not sufficient. Since the evaluation already supplies the future current profile, cutoff-crossing error can be assessed under that known profile without forecasting current. Please include this measure or narrow the related claims.

**********

what does this mean?). If published, this will include your full peer review and any attached files.

If you choose “no”, your identity will remain anonymous but your review may still be made public.

**Do you want your identity to be public for this peer review?**  For information about this choice, including consent withdrawal, please see our Privacy Policy

Reviewer #6: No

Reviewer #7: No

Reviewer #8: No

Reviewer #9: No

**********

[NOTE: If reviewer comments were submitted as an attachment file, they will be attached to this email and accessible via the submission site. Please log into your account, locate the manuscript record, and check for the action link "View Attachments". If this link does not appear, there are no attachment files.]

To ensure your figures meet our technical requirements, please review our figure guidelines: https://journals.plos.org/plosone/s/figures

You may also use PLOS’s free figure tool, NAAS, to help you prepare publication quality figures: https://journals.plos.org/plosone/s/figures#loc-tools-for-figure-preparation.

NAAS will assess whether your figures meet our technical requirements by comparing each figure against our figure specifications.

# Author comment: Author response to Decision Letter 3

**DOI:** 10.1371/journal.pone.0362362.r006

14 Oct 2026

Response Letter

We sincerely thank Editor-in-Chief, Associate Editor, and the reviewers for their constructive,

pertinent, and useful comments on our manuscript.

To Editor-in-Chief and Associate Editor

We are pleased to see insightful and constructive comments from the reviewers. In this resubmission, we have attempted to address the reviewers’ concerns and revised the manuscript

accordingly. Further details about all the changes we made can be found in our point-to-point

response to the reviewers’ comments below. In the revised manuscript, we marked the changes

in yellow.

To Reviewer 6

All the questions have been addressed well. I thank the authors for the prompt responses. I thus

recommend it for publication as is.

Response: Thank you so much.

To Reviewer 7

1. Tables 4,5 should be in the next page and increase the font like the journal guidline or event

your text.

Response: thank you for your comment. We have already used the largest possible font size while

ensuring that the tables fit within the page margins. Additionally, Tables 4 and 5 are positioned

close to their first mention in the text to maintain readability and coherence.

2. Some figures quality need to be improved and enlarge the size of figures while the font size of

axis and legends needs to be increased.

Response: Thank you for your comment. We have increased the font sizes of the axis labels, tick

labels, and other text elements in Fig. 8 to improve readability.

3. In equation 2, the matrix multiplication is dimensionally inconsistent.

Response: Thank you for your comment. We have corrected the matrix multiplication in Equation (2).

4. In equation 15, Separate symbols for V0 should be used.

Response: Thank you for your comment. We have revised Equations (11) and (15) to distinguish

the voltage parameters.

5. Ceff in equation 16 should be defined before Algorithm 1 or included among the required

decoder parameters.

Response: Thank you for your comment. We have revised Algorithm 1 to explicitly define the

effective capacity (Ceff) before the iterative ECM updates.

6. Strong cross-cell generalization, is strong without test temperature or other chemistry types,

rewrite in another words. Response: Thank you for your comment. We have revised the

statement to clarify that the model demonstrates promising predictive performance on unseen

battery cells within the evaluated datasets. The revised statement begins with "demonstrates

promising predictive performance...".

7. Remove such claims: Robust and reliable voltage predictions, and also more suitable for

real-world applications which is not adequately demonstrated because there is no embedded

implementation, real-time latency analysis.

Response: Thank you for your comment. We have revised the manuscript to remove unsupported

claims regarding robustness, reliability, and suitability for real-world applications. the revised

statement begins with "These results demonstrate that SeqBattNet achieves accurate terminal.."

8. Avoiding vanishing gradients should be replaced with sentence such as mitigating the vanishinggradient problem.

Response: Thank you for your comment. We have replaced "avoiding vanishing gradients" with

"mitigating the vanishing-gradient problem".

To Reviewer 8

1. R0, tau, SOC0, SOH, and vRC,0 are learned from the voltage-prediction objective, without

independent measurements or parameter identification. Fig. 6 shows that these quantities affect

the predicted voltage, but this does not establish that their learned values are physically correct

or uniquely identifiable. The description of these outputs as “physically meaningful parameters,”

particularly in Sections 5.3 and 6, should therefore be qualified.

Response: Thank you for your comment. We have revised Sections 5.3 and 6 to clarify that

Figure 6 demonstrates parameter sensitivity rather than physical accuracy or identifiability. The

revised statements begin with "These findings indicate that the encoder-generated..." in Section

5.3 and "The sensitivity analysis shows that..." in Section 6.

2. It is not fully clear how the five runs were used. Section 5.1 first states that their predictions

were averaged to obtain the final prediction, but later states that the reported metrics are the

averages of five independently calculated results. Please clarify which procedure was used in

Tables 4–6. As the five runs have already been completed, the standard deviations for the main

results could also be reported.

Response: Thank you for your comment. We have revised Section 5.1 to clarify the evaluation

procedure. The revised statement begins with "We use the default random-number initialization...". Since the standard deviations across the five runs are small, we have omitted them

from Tables 4–6 to avoid overcrowding the tables. Instead, Figure 8 presents the 95% confidence

intervals to illustrate the variability in the proposed model’s performance.

3. The reported MAPE values appear to require correction. Equation (22) includes multiplication

by 100, but Tables 4–6 seem to report the errors as fractions. For instance, an MAE of about

0.019 V for a voltage around 3–4 V would give a MAPE of roughly 0.5%, not 0.0055%. Please

check the calculation and the “MAPE (%)” headings throughout.

Response: Thank you for your comment. We have revised the MAPE definition in Equation (21)

to express the error as a fraction rather than a percentage. Accordingly, we have removed the

percentage symbol from the MAPE headings in Tables 4–6.

4. The literature on physics-integrated battery learning is still somewhat limited. Relevant

studies include but are not limited to “MINN: Learning the dynamics of differential-algebraic

equations and application to battery modeling” and “BMINN: Learning chemical potentials and

parameters from voltage data for multi-phase battery modeling.”

Response: Thank you for your comment. We have expanded the literature review in Section 1

to include the MINN and BMINN studies. The revised paragraph begins with "Recent studies

have further explored physics-integrated learning...".

5. There are still a few presentation issues. The RT-Batt heading repeats “Different Discharge

Profiles for cells.” The use of “Fig” and “Figure” is inconsistent, as is the capitalization in the

dataset headings. RMSE, MAE, and MAPE are also written with unnecessary spaces between

letters in several places.

Response: Thank you for your comment. We have corrected the duplicated RT-Batt heading,

standardized the capitalization of dataset headings and figure references, and fixed the spacing

of RMSE, MAE, and MAPE throughout the manuscript.

To Reviewer 9

1. The no-aging ablation uses parameters selected from initial cycles and then fixed, which

also changes the fitting procedure. Please clarify which parameters are fixed and whether the

remaining components are trained identically. This comparison supports the value of adaptation

but does not fully isolate its contribution.

Response: Thank you for your comment. We have revised Section 5.3 to specify the fixed

parameters and clarify that the remaining components retain the same training settings.

2. The explanation for omitting end-of-discharge timing error is not sufficient. Since the evaluation already supplies the future current profile, cutoff-crossing error can be assessed under that

known profile without forecasting current. Please include this measure or narrow the related

claims.

Response: Thank you for your comment. We agree that EOD timing error can be evaluated

under a known future current profile. As this study focuses on battery modeling and terminal

voltage prediction, we have revised Sections 1, 3.4, and 5.6 by adding the statements "The

proposed discrete-state formulation enables sequential terminal. . . ", "As this study focuses on

battery voltage modeling. . . ", and "This study focuses exclusively on battery modeling. . . ",

respectively, to clarify the study’s scope and avoid unsupported EOD/RDT performance claims.

Attachment Submitted filename: Response_to_Reviewers (1).pdf

# Peer review: Decision Letter 3

**DOI:** 10.1371/journal.pone.0362362.r007

**Authors:** Shih-Lin Lin

26 Oct 2026

SeqBattNet: A Discrete-State Physics-Informed Neural Network with Aging Adaptation for Battery Modeling

PONE-D-25-57156R3

Dear Dr. Trinh,

We’re pleased to inform you that your manuscript has been judged scientifically suitable for publication and will be formally accepted for publication once it meets all outstanding technical requirements.

Within one week, you’ll receive an e-mail detailing the required amendments. When these have been addressed, you’ll receive a formal acceptance letter and your manuscript will be scheduled for publication.

An invoice will be generated when your article is formally accepted. Please note, if your institution has a publishing partnership with PLOS and your article meets the relevant criteria, all or part of your publication costs will be covered. Please make sure your user information is up-to-date by logging into Editorial Manager at Editorial Manager® and clicking the ‘Update My Information' link at the top of the page. For questions related to billing, please contact billing support.

If your institution or institutions have a press office, please notify them about your upcoming paper to help maximize its impact. If they’ll be preparing press materials, please inform our press team as soon as possible -- no later than 48 hours after receiving the formal acceptance. Your manuscript will remain under strict press embargo until 2 pm Eastern Time on the date of publication. For more information, please contact onepress@plos.org.

Kind regards,

Shih-Lin Lin, Ph.D

Academic Editor

PLOS One

Additional Editor Comments (optional):

Reviewers' comments:

Reviewer's Responses to Questions

**Comments to the Author**

Reviewer #7: All comments have been addressed

**********

2. Is the manuscript technically sound, and do the data support the conclusions?

Reviewer #7: Yes

**********

3. Has the statistical analysis been performed appropriately and rigorously?

Reviewer #7: Yes

**********

4. Have the authors made all data underlying the findings in their manuscript fully available?

The PLOS Data policy

Reviewer #7: Yes

**********

5. Is the manuscript presented in an intelligible fashion and written in standard English?

Reviewer #7: Yes

**********

Reviewer #7: The authors have satisfactorily addressed my previous comments, and the manuscript has been revised accordingly. I have no further comments at this stage.

**********

what does this mean?). If published, this will include your full peer review and any attached files.

If you choose “no”, your identity will remain anonymous but your review may still be made public.

**Do you want your identity to be public for this peer review?**  For information about this choice, including consent withdrawal, please see our Privacy Policy

Reviewer #7: No

**********

# Editor report: Acceptance letter

**DOI:** 10.1371/journal.pone.0362362.r008

**Authors:** Shih-Lin Lin

PONE-D-25-57156R3

PLOS One

Dear Dr. Trinh,

I'm pleased to inform you that your manuscript has been deemed suitable for publication in PLOS One. Congratulations! Your manuscript is now being handed over to our production team.

At this stage, our production department will prepare your paper for publication. This includes ensuring the following:

* All references, tables, and figures are properly cited

* All relevant supporting information is included in the manuscript submission,

* There are no issues that prevent the paper from being properly typeset

You will receive further instructions from the production team, including instructions on how to review your proof when it is ready. Please keep in mind that we are working through a large volume of accepted articles, so please give us a few days to review your paper and let you know the next and final steps.

Lastly, if your institution or institutions have a press office, please let them know about your upcoming paper now to help maximize its impact. If they'll be preparing press materials, please inform our press team within the next 48 hours. Your manuscript will remain under strict press embargo until 2 pm Eastern Time on the date of publication. For more information, please contact onepress@plos.org.

You will receive an invoice from PLOS for your publication fee after your manuscript has reached the completed accept phase. If you receive an email requesting payment before acceptance or for any other service, this may be a phishing scheme. Learn how to identify phishing emails and protect your accounts at https://explore.plos.org/phishing.

If we can help with anything else, please email us at customercare@plos.org.

Thank you for submitting your work to PLOS One and supporting open access.

Kind regards,

PLOS One Editorial Office Staff

on behalf of

Professor Shih-Lin Lin

Academic Editor

PLOS One
